# Supplementary material for: Viability testing and transplantation of marginal livers (VITTAL) using normothermic machine perfusion: study protocol for an open-label, non-randomised, prospective, single-arm trial
Source: BMJ Open. 2017 Nov 28;7(11):e017733. doi: 10.1136/bmjopen-2017-017733 (PMC5719273; doi:10.1136/bmjopen-2017-017733)
Supplement: Supplementary file 1 [file bmjopen-2017-017733supp001.pdf]

Appendix 1. Definition of adverse events

ADVERSE EVENT (AE)

Any untoward medical occurrence in a subject.

NOTE this definition does not imply that there is a relationship between the adverse event and the device under investigation.

SERIOUS ADVERSE EVENT (SAE)

An adverse event that

a) led to a death,

b) led to a serious deterioration in the health of the subject that

1) resulted in a life-threatening illness or injury,

2) resulted in a permanent impairment of a body structure or a body function,

3) required in-patient hospitalization or prolongation of existing hospitalization,

4) resulted in medical or surgical intervention to prevent permanent impairment to body structure or a body function.

c) led to foetal distress, foetal death or a congenital abnormality or birth defect.

ADVERSE DEVICE EFFECT (ADE)

Any untoward and unintended response to a medical device.

NOTE 1 This definition includes any event resulting from insufficiencies or inadequacies in the instructions for use or the deployment of the device.

SERIOUS ADVERSE DEVICE EFFECT (SADE)

Adverse device effect that has resulted in any of the consequences characteristic of a serious adverse event or that might have led to any of these consequences if suitable action had not been taken or intervention had not been made or if circumstances had been less opportune.

UNANTICIPATED SERIOUS ADVERSE DEVICE EFFECTS (USADE)

Any serious adverse device effect which, by its nature, incidence, severity or outcome, has not been identified in the anticipated AE’s listed in section 8.1.1.

DEVICE DEFICIENCY

Inadequacy of a medical device with respect to its identity, quality, durability, reliability, safety or performance. Device deficiencies include malfunctions, use errors and inadequate labelling.

USE ERROR

Act or omission of an act that results in a different medical device response than intended by the manufacturer or expected by the user. Use error includes slips, lapses and mistakes. An unexpected physiological response of the subject does not itself constitute a use error.

SEVERITY DEFINITIONS

The following definitions will be used to determine the severity rating for all adverse events:
Mild: awareness of signs or symptoms, that does not interfere with the subject's usual activity or is transient that resolved without treatment and with no sequelae.
Moderate: a sign or symptom, which interferes with the subject's usual activity.
Severe: incapacity with inability to do work or perform usual activities.

Clavien Dindo Classification

Grades	*Definition*
Grade I:	Any deviation from the normal postoperative course without the need for pharmacological treatment or surgical, endoscopic and radiological interventions. This grade also includes wound infections opened at the bedside.<br>[Allowed therapeutic regimens are: drugs as anti-emetics, antipyretics, analgesics, diuretics and electrolytes and physiotherapy.]
Grade II:	Requiring pharmacological treatment with drugs (other than those noted above) including blood transfusions and total parenteral nutrition
Grade III:	Requiring surgical, endoscopic or radiological intervention
Grade III-a:	intervention not under general anaesthesia
Grade III-b:	intervention under general anaesthesia
Grade IV:	Life-threatening complication (including CNS complications)[1] requiring HDU/ICU management
Grade IV-a:	single organ dysfunction (includes dialysis)
Grade IV-b:	multi organ dysfunction
Grade V:	Death of a patient
Suffix 'd':	If the patients suffers from a complication at the time of discharge, the suffix “d” (for ‘disability’) is added to the respective grade of complication.

[1]brain haemorrhage, ischaemic stroke, subarachnoid haemorrhage, but excluding transient ischaemic attacks; HDU, high dependency unit; ICU, intensive care unit.
