# Supplementary material for: Viability testing and transplantation of marginal livers (VITTAL) using normothermic machine perfusion: study protocol for an open-label, non-randomised, prospective, single-arm trial
Source: BMJ Open. 2017 Nov 28;7(11):e017733. doi: 10.1136/bmjopen-2017-017733 (PMC5719273; doi:10.1136/bmjopen-2017-017733)
Supplement: Author's manuscript [file bmjopen-2017-017733.draft_revisions.pdf]

BMJ Open is committed to open peer review. As part of this commitment we make the peer review history of every article we publish publicly available.

When an article is published we post the peer reviewers' comments and the authors' responses online. We also post the versions of the paper that were used during peer review. These are the versions that the peer review comments apply to.

The versions of the paper that follow are the versions that were submitted during the peer review process. They are not the versions of record or the final published versions. They should not be cited or distributed as the published version of this manuscript.

BMJ Open is an open access journal and the full, final, typeset and author-corrected version of record of the manuscript is available on our site with no access controls, subscription charges or pay-per-view fees (http://bmjopen.bmj.com).

If you have any questions on BMJ Open's open peer review process please email editorial.bmjopen@bmj.com

BMJ Open

Viability testing and transplantation of marginal livers (VITTAL) using normothermic machine perfusion: An open label, non-randomised, prospective, single arm trial

Journal:	*BMJ Open*
Manuscript ID	bmjopen-2017-017733
Article Type:	Protocol
Date Submitted by the Author:	12-May-2017
Complete List of Authors:	Laing, Richard; University of Birmingham, National Institute for Health Research (NIHR) Birmingham Liver Biomedical Research Unit; University Hospitals Birmingham NHS Foundation Trust, Liver Unit<br>Mergental, Hynek; University Hospitals Birmingham NHS Foundation Trust, Liver Unit<br>Wilkhu, Manpreet; University of Birmingham, NIHR Liver BRU Clinical trials group (EDD), CRUK clinical trials unit<br>Barton, Darren; University of Birmingham, NIHR Liver BRU Clinical trials group (EDD), CRUK clinical trials unit<br>Curbishley, Stuart; University of Birmingham, National Institute for Health Research (NIHR) Birmingham Liver Biomedical Research Unit<br>Yap, Christina; University of Birmingham, NIHR Liver BRU Clinical trials group (EDD), CRUK clinical trials unit<br>Kirkham, Amanda; University of Birmingham, NIHR Liver BRU Clinical trials group (EDD), CRUK clinical trials unit<br>Boteon, Yuri; University of Birmingham, National Institute for Health Research (NIHR) Birmingham Liver Biomedical Research Unit; University Hospitals Birmingham NHS Foundation Trust, Liver Unit<br>Neil, Desley; University Hospitals Birmingham NHS Foundation Trust, Histopathology<br>Hubscher, Stefan; University Hospitals Birmingham NHS Foundation Trust, Histopathology<br>Perera, T; University Hospitals Birmingham NHS Foundation Trust, Liver Unit<br>Muiesan, Paolo; University Hospitals Birmingham NHS Foundation Trust, Liver Unit<br>Isaac, John; University Hospitals Birmingham NHS Foundation Trust, Liver Unit<br>Roberts, Keith; University Hospitals Birmingham NHS Foundation Trust, Liver Unit<br>Cilliers, Hentie; University Hospitals Birmingham NHS Foundation Trust, Liver Unit<br>Afford, Simon; University of Birmingham, National Institute for Health Research (NIHR) Birmingham Liver Biomedical Research Unit<br>Mirza, Darius; University Hospitals Birmingham NHS Foundation Trust, Liver Unit
**Primary Subject Heading**:	Surgery

Secondary Subject Heading:	Gastroenterology and hepatology
Keywords:	Hepatobiliary disease < GASTROENTEROLOGY, Hepatology < INTERNAL MEDICINE, Hepatobiliary tumours < ONCOLOGY, Transplant surgery < SURGERY, TRANSPLANT SURGERY

SCHOLARONE™ Manuscripts

For peer review only

Viability testing and transplantation of marginal livers (VITTAL) using normothermic machine perfusion: An open label, non-randomised, prospective, single arm trial

Richard W Laing[1,2*], Hynek Mergental[1,2*], Manpreet Whilku[3], Darren Barton[3], Stuart Curbishley[2], Christina Yap[3], Amanda Kirkham[3], Yuri L Boteon[1,2], Desley A Neil[1], Stefan G Hübscher[1,2], M Thamara P Perera[1,2], Paolo Muiesan[1,2], John Isaac[1], Keith J Roberts[1], Hentie Cilliers[1], Simon C Afford[2], Darius F Mirza[1,2].

1. Liver Unit, Queen Elizabeth Hospital, University Hospitals Birmingham NHS Foundation Trust, UK
2. National Institute for Health Research (NIHR) Birmingham Liver Biomedical Research Unit, Institute of Immunology and Immunotherapy, University of Birmingham, UK
3. CRUK Clinical Trials Unit. The University of Birmingham, UK

ADDRESS FOR CORRESPONDENCE
Professor Darius F Mirza, FRCS
Liver Unit, Queen Elizabeth Hospital Birmingham
University Hospitals Birmingham NHS Foundation Trust
Birmingham, B15 2TH
United Kingdom
Email: Darius.Mirza@uhb.nhs.uk
Tel: 0044 0121 371 4638
Fax: 0044 0121 414 1833

Word Count 7729

For peer review only

LIST OF ABBREVIATIONS

ALT, alanine transaminase;

AST, aspartate transaminase;

BAR, balance of risk score;

CIT, cold ischemic time;

CRCTU, Cancer Research UK Clinical Trials Unit;

DBD, donation after brain-stem death;

DCD, donation after circulatory death;

DRI, donor risk index;

eCRF, electronic case report form;

FWIT, functional warm ischemic time;

HMP, hypothermic machine perfusion;

HRA, Health Research Authority;

ICU, intensive care unit;

MHRA, Medicines and Healthcare Products Regulatory Agency;

MRCP, magnetic resonance imaging of the biliary and pancreatic tree;

NHSBT, National Health Service Blood and Transplant;

NMP, normothermic machine perfusion;

NMP-L, normothermic machine perfusion of the liver;

RINTAG, Research, Innovation and Novel Technologies Advisory Group;

SCS, static cold storage;

UHBFT, University Hospitals Birmingham NHS Foundation Trust;

VITTAL, Viability testing and transplantation of marginal livers.

For peer review only

ABSTRACT

Introduction

The use of marginal, or extended criteria donor livers is increasing. These organs carry a greater risk of initial dysfunction and early failure, as well as inferior long-term outcomes. As such, many are rejected due to a perceived risk of use and utilisation varies widely between centres. Ex-situ normothermic machine perfusion of the liver (NMP-L) has been shown to provide objective information on liver function and may enable safe transplantation of organs that are currently deemed untransplantable.

Methods and analysis

VITTAL (Viability testing and transplantation of marginal livers) is an open label, non-randomised, prospective, single arm trial designed to determine whether currently unused donor livers can be salvaged and safely transplanted with equivalent outcomes in terms of patient survival. The procured livers must be rejected for clinical use by all the UK centres and meet pre-defined criteria that objectively denote their marginal condition. The liver is subjected to NMP-L following a period of static cold storage. Organs metabolising lactate to ≤2.5mmol/L within 4 hours of the perfusion commencing in combination with two or more of the following parameters – bile production, metabolism of glucose, a hepatic arterial flow rate ≥150ml/min and a portal venous flow rate ≥500ml/min, a pH ≥7.30 and/or maintain a homogenous perfusion – will be considered viable and transplanted into a suitable consented recipient. The co-primary outcome measures are the success rate of NMP-L to produce a transplantable organ and 90-day patient post-transplant survival.

Ethics and dissemination

The protocol was approved by the National Research Ethics Service (London – Dulwich Research Ethics Committee, 16/LO/1056), the Medicines and Healthcare Products Regulatory Agency and is endorsed by the National Health Service Blood and Transplant Research, Innovation and Novel Technologies Advisory Group. The findings of this trial will be disseminated through national and international presentations and peer-reviewed publications.

Registration

Clinicaltrials.gov NCT02740608

For peer review only

Strengths and Limitations of the Study

- The study aims to establish objective liver viability criteria and biomarkers
- The viability testing of high-risk livers has the potential to significantly increase the number of transplantable organs
- The study has clearly defined criteria characterising the discarded organs
- The three-stage study design prioritises the patients' safety
- The trial includes low and moderate risk recipients – the suitability for high-risk recipients will require further testing

Keywords

Liver transplant, extended criteria donor, machine perfusion, normothermic, viability testing.

For peer review only

INTRODUCTION

Liver transplantation

Liver transplantation is a highly successful treatment for end stage liver disease, fulminant hepatic failure and early stage primary liver cancer. Deaths from liver disease have soared by 40 per cent in a decade and continue to rise. Liver disease kills 11,000 a year in England and the average age of death from liver disease (59 years), continues to decrease.[1] Over the past 50 years, transplant techniques and outcomes have greatly improved and 5-year survival rates of 70-80% mean that transplantation has become the mainstay of treatment for an increasing number of patients with chronic liver disease, metabolic disorders, acute liver failure and malignancy. As such, the demand for donor livers greatly exceeds supply and approximately 20% of patients die whilst awaiting transplantation.[2] In Europe, the most common indications for liver transplantation are cirrhosis (68%), malignancy (14%), and acute hepatic failure (8%). The main causes for cirrhosis in Europe are the hepatotropic viruses and alcohol related liver disease.[3] Non-alcoholic fatty liver disease is an emergent cause and despite health campaigns, the incidence continues to rise. In the UK, it is predicted that the incidences of end stage liver disease and hepatocellular carcinoma will increase substantially during the next decade, exacerbating the existing shortage of donor livers.

The UK Liver Transplant Programme

Between March 2015 and April 2016, there were 1161 new waiting list registrations in the UK, and 878 transplants were carried out. Of the 621 patients on the list as of April 2015, 22% died or were removed from the list (n=135) due to deteriorating health.[4] This is reflected across other countries to the extent that a patient is now more likely to die within the first 12 months of being listed than the first 12 months' post-transplant.[5] Over the past decade there has been a very modest increase in the use of standard or 'ideal' organ donors (those retrieved from young donors following a diagnosis of brain-stem death, DBD). In response, centres have utilised donors following circulatory death (DCD) and sub-optimal "marginal" or "extended criteria" donors (those of older age, livers with a presence of steatosis etc.).

Responding to the shortage

There are several ways to respond to the shortage. Organ donation policies are undergoing changes however there is a lack of well-controlled scientific evidence on which to base decisions regarding policy-making and opinions are strong and divided. Spain has the highest organ donation rates and operates an opt-out system, however the rise in rates only started

For peer review only

approximately 10 years after the system's introduction. Wales is the most recent country to go down this route, however unlike in Spain, next of kin consent is still required before patients can become organ donors. More likely, the increased Spanish donation rates are due to a combination of factors – the creation of a transplant coordination network that operates at hospital, regional and national levels, the placement of transplant coordinators at each procurement hospital and the improvement in the quality of information received by the public. Living donation is one potential means to increase the number of liver transplants, using surgical techniques developed for liver resection and 'liver splitting' (which uses a single liver for transplantation into two recipients). The major limitations are most patients do not have a willing or suitable living donor and there are concerns about the risks to the healthy donor. The reported risk of donor death is estimated at 0.2% but the risk of serious complications is much higher.[6 7] Although programmes have had some success in countries without deceased donor programmes, living donor transplantation will be unlikely to have a significant impact on the shortage of donor livers in most countries.

The use of "marginal" or "extended criteria" donors

As discussed, a rising proportion of transplants are carried out using "marginal" or "extended criteria" grafts, procured from obese or elderly donors with multiple co-morbidities.[8] These livers are significantly more susceptible to cold storage-related ischaemic injury, which increases the risk of graft failure, and recipient morbidity and mortality. Reflecting the issues with these sub-optimal grafts, in 2014/15, of 1282 solid organ donors, only 924 (72.1%) livers were deemed suitable for retrieval and only 812 (63.3%) were subsequently transplanted.[9] The duration of the functional warm ischemic time (FWIT) is an important determinant of outcome. The recent document 'Donation After Circulatory Death' published by a steering group on behalf of the British Transplantation Society and Intensive Care Society suggested that the stand-down time from the onset of functional warm ischaemia for DCD liver transplantation was 30 minutes (although 20 minutes is ideal), and that age was an important factor. Because of this, a number of livers will be retrieved from DCDs that fall into the "marginal donor" category and may not go on to be transplanted.[10]

Several donor parameters have been identified as relative risk factors for poor outcome including age; steatosis; DCD donation; split livers; prolonged cold ischaemia time (>12 hours). These were all developed using North American data and formulated into an algorithm known as the Donor Risk Index (DRI), and later validated using European data.[11 12]

For peer review only

The British Transplantation Society have published their own guidelines on the utilisation of donor organs and use criteria in Table 1 to distinguish between grafts of varying quality.

Table 1. Criteria for donor quality as per British Transplantation Society UK Guidelines for Donors after Circulatory death

Good livers – *All should be used* (DBDs and DCDs)	Ideal Livers – *All should be used* (DCDs)	Marginal Donors – *Use selectively* (DCDs)	Absolute contraindications to using liver as donor organ
• Age <50<br>• Normal LFTs<br>• <5 days on ICU<br>• Low levels of inotropic support<br>• <30% Steatosis<br>• No active sepsis	• Age <50 years<br>• Weight <100kg<br>• FWIT <20 mins<br>• CIT <8 hours<br>• <15% Steatosis<br>• ICU stay <5 days	• Age >50 years<br>• Weight >100kg<br>• FWIT 20-30mins<br>• CIT 8-12 hrs<br>• >15% Steatosis<br>• ICU stay >5 days	• DCD with macrosteatosis steatosis >30%<br>• ESLD<br>• Acute liver failure<br>• Acute liver injury that's not improving

CIT, cold ischemic time; DBD, donor following brain death; DCD, donor following circulatory death; ESLD, end-stage liver disease; FWIT, functional warm ischemic time; ICU, intensive care stay; LFTs, liver function tests

For peer review only

Organ Preservation

The current standard of donor liver preservation is based on static cold storage (SCS).[13] During SCS, organs are flushed and cooled with specific chilled preservation solutions (University of Wisconsin [UW] solution is used most commonly although Histidine-Tryptophan-Ketoglutarate (HTK) solution is also used less widely) and ice is added to the abdominal cavity. After retrieval, the organ is placed in fluid-filled sterile plastic bags for transportation and stored in preservation solution within an ice-box until transplantation. Although the available preservation solutions differ in chemical composition, their function is essentially the same. The hypothermia aims to reduce the liver's metabolic activity and the solution aims to reduce the cellular swelling. This is a consequence of anaerobic metabolism resulting in depletion of adenosine triphosphate stores leading to influx of free calcium and activation of phospholipases.[14] Cooling the organ slows metabolism approximately 12-fold but cannot prevent its dysfunction and the eventual destruction of cellular integrity. Ischaemia-reperfusion is an important factor influencing graft outcome.[15] The ischaemic phase starts early in the procurement process (swings in blood pressure following brain-death or due to the functional warm ischaemic time in non-heart beating donors) and triggers a complex cascade of cellular and molecular events including the release of pro-inflammatory

mediators and chemotaxis of cell types that initiate progressive immunological processes. During the reperfusion phase, "the reflow paradox" causes infiltration of the tissues by leucocytes and cellular injury occurs through a series of pathways that include lipid peroxidation and the creation of reactive oxygen species[16] The most common manifestation of the ischaemia-reperfusion process is delayed graft function, which is the inability of the organ to fulfil the physiological needs of the recipient and is associated with graft failure, re-transplantation and death.[17] Static cold storage therefore is unable to reverse the injury sustained during donor death and procurement, causes injury due to the cooling process, limits the preservation time and prevents physiological assessment prior to transplantation.

In-situ organ reconditioning

To reverse or diminish the injury, many cytoprotective strategies have been tested in experimental models of transplantation and several have been shown to have therapeutic potential, including gene therapy,[18] [19] cytokine or growth factor administration,[20-22] vasodilating agents and ischemic pre-conditioning.[23] [24] Treatment of the organ during preservation has major logistic and ethical advantages over any attempt to achieve the same effects by treating the donor (therapeutic interventions before declaration of death are not currently permitted unless they are of potential benefit to the donor). Recently there has been published early experience with normothermic regional perfusion of DCD donors, nevertheless the feasibility and benefit of this experimental approach is yet to be shown.[25]

Normothermic Machine Perfusion of the Liver (NMP-L)

Bretschneider and Starzl first attempted machine perfusion of the liver in the late 1960's. Although hypothermic machine perfusion (HMP) has been shown some promise in clinical studies, NMP-L combats the limitations of SCS previously described by aiming to maintain the organ at the body's natural temperature while providing oxygen, nutrition and the essential substrates necessary for adequate cellular metabolism. Providing a homeostatic environment theoretically enables us to extend our storage period and test the organs physiological parameters. To date only one clinical trial of 20 adult recipients of livers maintained by HMP has been published showing a reduction in early graft dysfunction (5% vs 25% $p<0.08$) as well as a significant reduction in serum injury markers in the HMP group. A joint pilot trial between Oxford University, King's College Hospital London and University Hospitals Birmingham Foundation Trust (UHBFT) recruited 20 patients into a NMP-L phase 1 study and concluded the procedure was feasible and safe when used on

For peer review only

current conventional donor acceptance criteria.[26] Following this, a 220-patient phase III international clinical trial entitled "COPE WP2" has completed recruitment and the results are eagerly awaited. The Liver Unit at University Hospital Birmingham NHS Foundation Trust contributed to this multi-centre international trial by randomising 50% of the study patients.

Our group believes NMP-L enables the donor organ to be functionally assessed, thereby increasing transplant safety. It can also extend organ preservation times to improve transplant logistics and donor organ utilisation. There are several devices available on the market, but only the OrganOx *metra* ™ has been widely used in the clinical transplant setting.[26] Our team has performed over 70 liver transplants with grafts preserved on this machine and has gained broad experience by using this device. The OrganOx *metra* ™ is the leading device in terms of the number of clinical transplants undertaken, with more than 100 machine-perfused livers transplanted in the Phase III randomised European trial, together with 20 livers in the Phase I safety study and further on-going trials in North America. For these reasons, we have decided to use the OrganOx *metra* ™ device for the proposed study.

The device consists of a unit that cradles the liver, a perfusate reservoir, oxygenators, pumps operating at physiological pressures and a closed tubing system that connects the unit to the portal vein, hepatic artery and vena cava. The constituents of the perfusate can vary but generally consist of whole blood for oxygen carriage, sources of nutrition (glucose, insulin, amino acids), anti-thrombotic agents (heparin, epoprostenol), antibiotics and acid-base agents which help reduce cellular oedema, cholestasis, microvascular injury and the effects of free-radicals.

Benefits of NMP-L

NMP-L does not simply benefit marginal DCD organs that have been exposed to a damaging FWIT. Brain-stem death is a catastrophic physiological event associated with profound hypotension (parasympathetic response) followed by hypertension, tachycardia and high levels of circulating catecholamines (sympathetic surge) followed by another reduction in the sympathetic outflow. These dramatic swings can cause significant graft ischaemia prior to retrieval. Diabetes insipidus occurs in 70-80% of brain dead patients causing severe hypernatremia (associated with primary liver graft non-function), hypokalaemia, hypocalcaemia, hypophosphatemia and hypomagnesaemia.[27 28] Pirenne *et* al described seven

For peer review only

cases when livers from DBD's between 70 and 80 years old were used with "favourable outcomes".[29] More recently, groups from Italy have reported excellent outcomes using grafts from octogenarian donors.[30 31] NMP-L could however play an important role in preconditioning and assessing such organs prior to transplantation.

Hellinger et al were unable to identify a benefit using NMP-L in 1997; however, it was the first study of its kind.[32] In 2001, Schon used NMP-L to preserve and re-condition livers that had been exposed to 1 hour of warm ischaemia. These livers were then transplanted into pigs which all survived longer than 7 days. The group that received livers preserved using SCS had no survivors.[33] Several studies have been published by the Oxford group, responsible for OrganOx *metra*™. Imber *et al* published results from a study on a porcine model comparing NMP-L with SCS controls. They showed livers preserved using NMP-L were significantly superior (P<0.05) to SCS livers "in terms of bile production, factor V production, glucose metabolism, and galactose clearance", whilst SCS livers had higher perfusate levels of hepatocellular enzymes and more cellular damage.[34] The same year they successfully perfused and maintained 5 porcine livers for 72 hours, managing to maintain normal physiological parameters, pH, protein synthesis and histological architecture.[35] In 2009, Brockman et al simulated DBD and DCD scenarios in a porcine model. After five hours of preservation (NMP-L vs SCS) there was no difference seen in preservation method in either the DCD or DBD graft recipients. After 20 hours of preservation however, both DCD and DBD grafts that had been preserved using NMP-L were superior to their SCS counterparts with respect to enzyme release, histological changes and recipient survival. Of note, there was no difference in survival between DCD and DBD NMP-L-preserved graft recipients (83% and 86% respectively).[36]

Pre-clinical research and pilot study

Our team's pre-clinical research on rejected human livers has demonstrated that metabolism of lactate, in combination with bile production, maintenance of physiological pH, and stable blood flow rates, are sensitive parameters predictive of organ viability. In April 2014, the UHBFT Novel Therapeutics Committee approved a pilot clinical project for transplantation of five reconditioned liver grafts, initially deemed unusable for transplantation. In this series, livers were declined by all the UK transplant units, after which NMP-L commenced following a variable period of SCS. Still, five out of six tested livers met the viability criteria and were successfully transplanted.[37] Although this pilot project showed that viability testing

For peer review only

has the potential to transform the organ selection and acceptance process of high-risk livers, our observation primarily provided the feasibility and short-term outcome data. In addition, this cohort also demonstrated the feasibility of performing NMP-L within a "back-to-base" model, i.e. following SCS and inspection at the transplant centre. This offers logistical and financial advantages over using NMP-L in place of SCS and may target livers that would benefit the most from NMP-L. More research in this area is required and this was recognised by the Health Innovation Challenge Committee of the Wellcome Trust who awarded our study group a research grant to fund this trial. We have demonstrated so far, that a proportion of currently rejected liver allografts might be salvaged by subjecting them to NMP-L and viability testing. Use of this technology could transform the utilisation of high-risk organs and may improve access to treatment for thousands of patients awaiting liver transplantation globally.

METHODS

Study Design Overview

VITTAL is a phase II non-randomised, prospective, single-arm trial, performing NMP-L at a single site (UHBFT), on up to 53 grafts rejected for transplantation until 22 resulting clinical transplants are performed. It is designed using two linked components to assess both (A) the feasibility of NMP-L as a technique to increase the number of transplantable livers using a 2-stage design[38] and (B) Achievement of successful transplantation using rejected liver grafts. This will be assessed using a 3-stage design and measured by 90-day patient survival – a nationally accepted, monitored and continuously audited outcome following liver transplantation.[39]

Ethical and regulatory approval

The National Research Ethics Service (NRES) London-Dulwich (REC reference 16/LO/1056, Protocol number RG 15-240) and the Medicines and Healthcare Products Regulatory Agency (MHRA) approved all versions of the study protocol. This trial will use the OrganOx *metra* ™ device following a variable period of SCS to evaluate organ viability pre-transplant procedure. The OrganOx *metra* ™ device currently has a CE mark for liver organ transport and not organ evaluation. The use of the device within this clinical trial is therefore off registration and UK Competent authority (MHRA) clinical trial authorisation was obtained. In addition, approval from the Research and Development (R&D) department at UHBFT and from NHSBT's RINTAG was obtained prior to the start of screening.

For peer review only - http://bmjopen.bmj.com/site/about/guidelines.xhtml

Graft entry into study and subsequent preparation

The patient and donor liver pathways can be seen in Figure 1. All livers will be retrieved with the intention and standardised technique to use them for transplantation. Following the retrieval procedure at the donor hospital the liver will be placed in ice-cold preservation solution on the back-table and transported (according to local protocol). If the liver is allocated to UHBFT, if it is then considered not suitable for use it must be rejected by the on-call transplanting surgeon. For the liver to be considered untransplantable, the liver will be inspected by the on-call transplant surgeon and another transplant surgeon in the department. The liver will then be offered as a Fast Track graft to the other centres around the UK. If rejected by all centres and if consent for research was taken, it will be considered for use in VITTAL. Livers offered to our unit as Fast Track offers from other centres will undergo the same 2-consultant rejection process. An appropriate consented potential recipient will be selected by the transplant surgeon and contacted by the coordinator and will come into hospital for admission. The co-ordinator will request 3 units of packed red blood cells, matched to the intended recipient, for use in the OrganOx *metra*™ device. The liver will be prepared according to the procedure for preparing the device for use and placing the organ on the device (described in detail in the OrganOx *metra*™ Instructions for Use (IFU) document (version 13.0, 12-Mar-2016). The liver will be weighed prior to being connected to the device. If cannulation proves impossible, the liver will be rejected as previously intended. If the liver meets the criteria for transplantation, the recipient explantation will commence and the procedure for removing the liver from the device is also described in the IFU. Implantation and reperfusion of the liver will proceed as per the usual practice of the implanting centre. The patient will be clerked as if they were being admitted for a standard liver transplant.

Perfusion of the graft

The machine will be primed with a perfusate suitable for NMP-L and will use packed red cells as the oxygen carrier. During the perfusion, biochemical analysis of the blood-based perfusate will be performed using a Cobas biochemical point-of-care analyser (Roche Diagnostics) which will give results for pH, pO2, pCO2, Bicarb, Base excess, Calcium, Chloride, Sodium, Potassium, Haemoglobin, Haematocrit, Lactate and Glucose. Arterial and portal venous flows, resistances and pressures will also be recorded. Samples to be collected are detailed in Table 2.

For peer review only

For peer review only

Table 2. Trial sample collection schedule.

Perfusate samples	Hepatic arterial and hepatic venous biochemistry (point-of-care)	Pre-perfusion<br>Every 30 minutes during perfusion	Cobas point-of-care desktop analyser
	Perfusate supernatant	Pre-perfusion<br>Every 15 mins for first hour<br>Every hour thereafter	5x1ml aliquots<br>Stored at -80°C
Liver samples	Liver biopsy	L1 Pre-perfusion<br>L2 After 4 hours<br>L3 at end of perfusion*<br>L4 Post-reperfusion	16G core needle biopsy<br>Divided into segment for formalin, segment for frozen and piece for electron microscopy.
	Common bile duct	CBD1 Pre-perfusion<br>CBD2 Post-reperfusion	Formalin
Bile samples	(if produced)	B2 sample at 2 hours<br>B4 sample at 4 hours<br>B6 sample at 6 hours	Total volume recorded and 2ml samples snap frozen at these time points
Patient Samples	Biochemistry<br>Haematology<br>Clotting	Visits 1, 2, 3, 4, Extended follow-up	Standard of care
	Serum, Plasma, mononuclear cells (PBMC)	Visit 1 (pre-operative [post-induction of anaesthesia], post-reperfusion<br>Day 4 post-op)<br>Visits 2, 3, 4,	Additional research samples
	Urine	Visit 1 (pre-operative [post-induction], post-reperfusion<br>Day 4 post-op)	Additional research samples

*if lasting longer than 6 hours

For peer review only

The duration of machine perfusion will be dictated by logistics and the recipient's explant, but should not be less than 4 hours or more than 24 hours. For a graft to be considered for transplantation it *must* –

- Metabolise lactate to less than or equal to 2.5mmol/L within 4 hours of the start of the perfusion

And meet at least 2 of the following criteria within 4 hours of the start of perfusion:

- Demonstrate evidence of bile production
- Maintain a pH greater than 7.30
- Show evidence of glucose metabolism
- Maintain stable hepatic arterial flow of more than or equal to 150 mL/ minute and portal flow more than or equal to 500 mL/minute
- Achieve homogeneous graft perfusion with soft consistency of the parenchyma

Once the transplanting surgeon is content that the liver has met the criteria required for transplantation, the recipient will be brought to theatre and the explant will commence.

Explantation, implantation and reperfusion of the liver will be carried out in using standardised techniques by the on-call transplant surgeon. The liver will remain on the machine until after the explantation has taken place at which point it will be flushed by 2L of cold HTK immediately prior to implantation.

Concomitant therapy/medications

Patients will receive immunosuppression according to hospital protocols and other medications as necessary for their co-morbidities and current clinical condition. Their post-operative care will be the same as if they had undergone a standard liver transplant.

Outcome measures

Primary

There are 2 linked primary objectives and respective outcome measures.

Primary Objective (A) - Establish the feasibility of NMP-L to increase the number of transplantable livers.

For peer review only

Primary Outcome measure (A) – "Rescue rate" i.e. the proportion of rejected livers that can be used for transplantation having been deemed viable following a period of machine perfusion. As per study design, NMP-L is feasible if ≥22/53 perfusions of rejected liver grafts result in transplantation.

Primary Objective (B) – To achieve successful transplantation of previously rejected donor livers following viability testing using NMP-L.
Primary Outcome Measure (B) – 90-day patient survival, calculated as the number of patients alive 90-day post NMP-L treated marginal liver transplantation (numerator) divided by the total number of NMP-L treated marginal liver transplants performed, assessed at the end of the trial period at the final assessment point. The results will be compared to a matched group of recipients who have been transplanted with "transplantable" marginal donor livers at our centre. Retrospective data will be taken from a prospectively maintained database.

Secondary
No hypotheses testing is planned for any secondary outcome measures, and analyses will be descriptive only.
The assessment of graft function post-transplantation by incidence of primary non-function and early allograft dysfunction will be carried out by comparing results with a contemporary matched recipient group of patients obtained from a prospectively maintained database. The contemporary matched recipient group will be matched using the following:

- Patient Characteristics: Age, Sex, BMI, MELD, UKELD, Aetiology
- Donor Liver Characteristics: DCD or DBD, Sex

Secondary objective (A) – Assessment of liver graft function following transplantation (by incidence of primary non-function, and early allograft dysfunction)
Secondary Outcome measures (A) – Liver function tests; 90-day graft survival; 12-month patient and graft survival.

Secondary objective (B) – Assess morbidity associated with receipt of extended criteria graft that had previously been rejected.
Secondary Outcome measures (B) – Adverse event rates and severity, graded according to the Clavien-Dindo classification[40] (Appendix 1); Requirement of renal replacement therapy; Incidence of biliary complications (including incidence of ischemic type biliary lesions

For peer review only

diagnosed on MRCP at 6 months); Incidence of vascular complications; Biopsy-proven acute rejection; Reoperation rate; Length of intensive therapy unit stay; Length of hospital stay.

Secondary objective (C) – Assess the physiological response to reperfusion of the perfused grafts:

Secondary Outcome measures (C) – Post-reperfusion syndrome (Defined as a decrease in mean arterial pressure (MAP) of more than 30% from the baseline value for more than one minute during the first five minutes after reperfusion (assessed in the context of inotrope use))

Secondary objective (D) – Identify impact upon quality of life after transplantation with these liver grafts.

Secondary Outcome measures (D) – Quality of life by delivery of the EQ-5D-5L questionnaire

at baseline, day 30 and 6 months' post-transplant.

Analytical methods

Histopathology

Two independent liver histopathologists from UHBFT will perform all the histopathological assessments. Both will be blinded to the graft type, and the primary and secondary outcome measures although the presence or absence of a post-reperfusion biopsy means they will know whether a graft has met the criteria for transplantation. The histological analysis will be established using haematoxylin and eosin at two levels as well as, periodic acid Schiff (PAS), periodic acid Schiff diastase (PASD), haematoxylin van Gieson (HVG), reticulin, orcein, rhodanin and Perls stains of formalin-fixed paraffin-embedded liver tissue.

Perfusion, clinical and laboratory data

Donor and patient demographics as well as intraoperative data will be collected. BMI was defined as weight in kilograms divided by the square of the height in metres (kg/m^2). In non-heart beating (DCD) donation, FWIT is defined as the time between the systolic blood pressure of the donor dropping below 50mmHg until the point of aortic perfusion. Cold ischemic time is defined as the time between aortic perfusion and the start of NMP-L. Donor risk index (DRI) and Balance of Risk (BAR) will be calculated as per the relevant literature.[11] [41]

For peer review only

The perfusate fluid will undergo point-of-care biochemical testing every 30 minutes as previously described. Perfusate will be taken at the time points described in Table 2 and tested for transaminase, urea, albumin and factor V levels. Patient's blood samples will be analysed for full blood count, urea, creatinine and electrolytes, liver function tests, international normalised ratio (INR), prothrombin time, amylase, C reactive protein and plasma glucose using standard laboratory methods (Roche Modular system, Roche Ltd, Lewes, UK) both pre-and post-operatively. Research recipient blood and urine samples will also be taken as part of work package 2 (WP2) that will enable immune cell profiling as well as lipodomic, proteomic and metabolomic testing.

Patient questionnaires

Quality of life (QoL) will be assessed by delivery of the EQ-5D-5L questionnaire (UK (English) © 2009 EuroQol Group EQ-5D™ is a registered trademark of the EuroQol Group) at baseline, day 30 and 6 months' post-transplant. EQ-5D-5L is a 5-level version of the EQ-5D descriptive system (M. Herdman et al. Qual Life Res DOI 10.1007/s11136-011-99031). The 5L retains the 5-dimensional (5D) structure of the original EQ-5D-3L but the levels on each dimension were expanded to 5 based on qualitative and quantitative studies conducted by the EuroQol Group. Index-based values ('utilities') enable the calculation of quality-adjusted life years (QALYs) which help inform economic evaluations of health care interventions.

Statistical justification and outcome analysis

Sample size justification

For primary objective (A) (feasibility of NMP-L to rescue discarded liver grafts), it is anticipated that NMP-L will achieve a desirable organ recovery rate of at least 50%, with an undesirable rate of 30% or less as this would not be considered economically feasible. The significance level (α) is set at 0.05, corresponding to the probability of incorrectly rejecting the hypothesis given it is true (Type I error), and the power is set at 0.90 (Type II error rate, $\beta = 0.10$), corresponding to the probability of correctly deciding the NMP-L treatment is successful given the true response rate is greater than 50%. Using a Simon's two-stage design;[38]

Stage 1A of accrual: 24 marginal grafts will be perfused and assessed in the first stage. Grafts will be transplanted depending on the criteria achieved. The procedure will be considered

For peer review only

infeasible if there are fewer than 8 recovered livers. If more than 8 livers are transplanted, we will proceed to Stage 2A.

Stage 2A of accrual: Up to additional 29 marginal grafts will be perfused. We would consider the procedure feasible if there are at least 22 recovered livers out of 53 perfused livers.

For primary outcome (B), for viable livers transplanted following NMP-L, a desirable 90-day patient survival rate is at least 88%, with an undesirable rate of 73% (15% lower). The mean 90-day patient survival rate for 'standard' liver transplants is 93%.[42] An optimal three-stage design[39] will be used to test the null hypothesis that the mean 90-day patient survival rate will be less than 73% ($P \leq 0.73$), versus an alternative hypothesis - that the 90-day patient survival rate will be at least 88% ($P \geq 0.88$). The significance level is set at 0.20 (target $\alpha=0.2$), giving a 0.2 probability to conclude that a single transplantation is viable when it truly is not viable. The power is set at 80% (target $\beta=0.2$), giving a 0.2 probability to conclude that a single transplantation is not viable when it truly is viable;

Stage 1B: Following transplantation in 3 patients, the trial will stop early (concluding $P \leq 0.73$) if there are fewer than 2 patients achieving 90-day survival. If 2 or more patients reach the primary end point of 90-day survival, an additional 8 transplantations will be performed.

Stage 2B: Following transplantation in 11 patients (combined first and second stages) the trial will stop early (concluding $P \leq 0.73$) if there are seven or fewer successes. If 8 or more patients reach the primary end point, an additional 11 transplantations will be performed.

Stage 3B: Following transplantation in 22 patients in all three stages, the trial will be successful if at least 18 patients reach the primary end-point of 90-day survival.

The trial schema is provided in Figure 2.

Analysis of outcome measures

Primary Analysis

To assess (A), the feasibility of NMP-L, the rescue rate will be calculated as the number of perfused marginal grafts meeting the criteria for viability (numerator) divided by the total number of perfused marginal grafts (denominator).

$$Rescue\ Rate = \frac{Number\ of\ viable\ perfused\ marginal\ grafts}{Total\ number\ of\ perfused\ marginal\ grafts}$$

For peer review only

To assess (B) – achievement of successful transplantation of previously rejected donor liver following viability testing using NMP-L – we will evaluate 90-day patient survival rate, as an indicator of liver function and/or viability following transplantation of marginal liver grafts following NMP-L. The 90-day patient survival rate will be calculated as the number of patients alive 90-day post-transplant with a VITTAL graft, divided by the total number VITTAL-patients transplanted, assessed at the end of the trial period after the final assessment point (6 months OPD and MRCP).

$$90\ day\ patient\ survival\ rate = \frac{Number\ of\ patients\ alive\ at\ 90\ days\ post\ transplantation}{Total\ number\ of\ transplanations\ performed}$$

For (A), all livers undergoing NMP-L treatment will be included for evaluation in the interim and final analyses. For (B), all transplantations performed will be included for evaluation in the interim and final analyses. The rate outcomes will be reported together with confidence intervals using the Wilson (1927) method.[43]

Secondary Analysis

For all secondary outcome measures, analyses will be mainly descriptive. Continuous exploratory measures will be summarised via means, medians, standard deviations and ranges. Categorical measures will be summarised with number and proportion in each category. To model repeated measures over time (e.g. quality of life), a linear mixed effects model (considering subject correlation) using parametric and more flexible models may be considered. Time to event outcomes will be assessed using the method of Kaplan and Meier. Median survival with corresponding 95% confidence interval will also be reported where appropriate.

Conduct of trial

Donor liver selection

Suitable donor liver grafts will be selected from October 2016. Grafts will be retrieved with the intention to transplant and rejected as previously described.

Graft inclusion criteria

For peer review only

Rejected donor liver grafts must meet *all* of the following inclusion criteria to be eligible for inclusion in the VITTAL trial;

- Liver from a donor primarily accepted with the intention for clinical transplantation
- Rejected by all the other UK transplant centres via normal or fast-track sequence
- Cold ischaemic time less than 16 hours for DBD and 10 hours for DCD grafts
- One of the following parameters which would denote the marginal condition of the liver
    - Donor risk index greater than 2.0[11]
    - Graft macrovesicular steatosis greater than 30%
    - BAR score greater than 9[44]
    - Donor warm ischaemic time greater than 30 minutes
    - Anticipated cold ischaemic time greater than 12 hours for DBD or 8 hours for DCD liver grafts
    - Suboptimal liver graft perfusion documented by a photo of macroscopic appearance
    - Donor transaminases (ALT or AST) above 1000 IU/mL

Graft Exclusion Criteria

Livers meeting any of the following criteria would not be suitable for the VITTAL trial:

- Grafts from patients with active Hepatitis B, C or HIV infection
- Livers with macroscopic appearance consistent with cirrhosis
- Livers with advanced fibrosis
- DCD grafts with donor warm ischaemic time (systolic blood pressure less than 50mmHg to aortic perfusion) more than 60 minutes
- Excessive cold ischaemic times (DBD more than 16 hours / DCD more than 10 hours)
- Paediatric donor (<18 years)
- ABO incompatibility

Recipient Inclusion Criteria

Suitable potential VITTAL graft recipients will be identified during the listing process. Patients will be told that they are potentially suitable to receive a graft from the VITTAL trial and will be given the patient information sheets to read more about the trial. If already listed, potential recipients will be identified on the list, contacted and sent the same documentation.

For peer review only

If they wish to take part a minimum of verbal consent will be taken. Enrolling in the trial will in no way impact upon the chance of them receiving a standard 'transplantable' graft. Listed patients must meet all of the following inclusion criteria to be eligible for participation in the VITTAL trial:

- Adult primary liver transplant recipient
- Patient listed electively for transplantation
- Low to moderate transplant risk candidate, suitable for marginal graft, as assessed by the UHBFT liver transplant listing MDT meeting (these are usually candidates with low UKELD score, without cardiovascular comorbidities, with good functional and nutrition status, with patent portal vein and with no history of previous major upper abdominal surgery, e.g. patients transplanted for liver cancer)

Recipient Exclusion Criteria

Subjects who meet *any* of the following exclusion criteria are excluded from participating in the VITTAL trial:

- "High-risk patients" and recipients not considered suitable for a marginal graft (these are mainly patients with high UKELD score (>62 as per the NHSBT LAG criteria for graft sharing in high risks recipients in the North East of the UK with cardiovascular comorbidities or renal insufficiency, with poor nutrition and performance status or history of major upper abdominal surgery, e.g. patients listed for liver re-transplantation) [http://www.odt.nhs.uk/ search "Liver Allocation Policy"]
- Patients with complete portal vein thrombosis diagnosed prior to the transplantation
- Liver re-transplantation
- Patients with fulminant hepatic failure
- Patients undergoing transplantation of more than one organ
- Contraindication to magnetic resonance imaging (i.e. pacemaker fitted)

Adverse events reporting and analysis

The collection and reporting of Adverse Events (AEs) will be in accordance with the Research Governance Framework for Health and Social Care and the requirements of the National Research Ethics Service (NRES). Definitions of different types of AE are listed in (Appendix 1). The reporting period for AE's will commence at visit 1 and end at the 24-month follow-up. The Investigator should assess the seriousness and causality (relatedness)

For peer review only

of all AE's experienced by the patient (this should be documented in the source data) with reference to the protocol. This will include abnormal laboratory findings which are reported as clinically significant. All AE's, device deficiencies and ADE's will be reported using the applicable eCRF form. AE's will be reported in accordance with Clavien-Dindo classification of surgical complications[40]. Anticipated AE's include those related to any form of major surgery; infection (chest, urine, blood, bile, wound, abdominal), fluid collection (abdominal, pleural), renal dysfunction, cardiac failure, respiratory failure, and those related to the disease process and transplantation; early allograft dysfunction, rejection, hospitalisation for pre-existing condition that has not deteriorated, clinically significant abnormal laboratory finding or other abnormal assessments that is associated with the condition being studied (unless judged by the investigator as more severe than expected for the patient's condition). The investigator will exercise his/her medical judgment in deciding whether an abnormal laboratory finding or other abnormal assessment is clinically significant. However, if in the opinion of the investigator, the frequency or severity of the event is greater than would be expected then it must be reported. Device deficiencies that did not lead to an adverse event, but could have led to a medical occurrence if suitable action had not been taken, or intervention had not been made or if circumstances had been less fortunate, will also be recorded and reported.

Those events not being reported

The following are considered routine during or after liver transplantation and will not be reported as AE's.

- Initial admission to Intensive Care following liver transplant
- Elevation of AST and/or ALT <2000 iu/ml within 48 hours of liver transplant
- Transfusion of ≤ 5 units of packed red cells
- Transfusion ≤ 8 units of fresh frozen plasma
- Transfusion ≤ 2 adult doses of platelets

In addition to the above, medical and scientific judgement should be exercised in deciding whether expedited reporting is appropriate in other situations, such as important medical events that may not be immediately life threatening or result in death or hospitalisation but may jeopardise the patient or may require intervention. Any death occurring during the protocol defined follow up period (within 90 days), whether considered device-related or not,

For peer review only

must be reported as an SAE within 24 hours of the local investigator becoming aware of the event. If a death occurs in a patient receiving a transplant the cause of death will be investigated and reviewed by the Trial Management Group (TMG) and clinical team caring for the patient. Entry of patients in to the study would be temporarily suspended until these investigations are complete.

Study visit overview

The VITTAL trial involves a minimum of four patient visits which all coincide with standard admissions either for surgery or for outpatient follow-up. There are no additional trial-specific visits. The schedule for the study visits and data collection is summarised in table 3. Visit 1 encapsulates admission for transplant and the post-operative period if the transplant proceeds. Visits 2, 3 and 4 are scheduled for 30 day, 90 day and 180 day follow-up respectively. All patients will undergo MRCP during visit 4 to investigate the occurrence of ischemic-type biliary lesions which also marks trial end-point. Patients will continue to be followed up at 12 months and 24 months as part of their standard post-transplant care and data will be collected at these time-points for long-term reporting.

For peer review only

Table 3: Patient Schedule of Events

Patient Registration	**Screening**	**Visit 1 Transplant** Day 0	**Visit 2** Day 30 (+/- 3days)	**Visit 3** Day 90 (+ 3 days)	**Visit 4** Day 180 (+ 30 days)	**Extended follow up** 12 month + 24 month (+/- 30 days)
Informed consent	X					
Eligibility assessment	X	X				
Patient history	X	X				
Standard routine blood tests*	X	X	X	X	X	X
MELD (automatically calculated)		X				
UKELD (automatically calculated)		X				
Trial specific additional patient samples blood and urine		X	X	X	X	
PBMC Collection		X	X	X	X	
Liver Biopsy 4 (see table 2)		X				
Quality of Life questionnaire (EQ-5D-5L)		X	X		X	
Patient Resource Log at Visit 1 discharge		X				
Adverse/ Clinical events	X	X	X	X	X	X
Concomitant medications	X	X	X	X	X	X
MRCP					X	

* Standard routine blood tests - Full blood count (FBC), urea, electrolytes, liver function tests, AST, GGT, eGFR, international normalised ratio (INR)

For peer review only

Storage of samples

Patient blood samples taken as part of their standard of care will be processed and stored according to UHBFT procedures. Perfusate, patient serum, plasma, urine samples and mononuclear cell preparations collected during visits 1-4 will be stored frozen in 0.5–1.0 mL aliquots at – 80°C at the Institute of Biomedical Research, University of Birmingham. Liver biopsy tissue specimens will be collected and the formalin fixed paraffin embedded segments will be processed by staff in the department of cellular pathology at UHBFT. After sectioning and staining, tissue blocks will be stored at the Institute of Biomedical Research. All samples will be collected in accordance with national regulations and requirements including standard operating procedures for logistics and infrastructure. Samples will be taken in appropriately licensed premises, stored and transported in accordance with the Human Tissue Authority guidelines and trust policies.

Data handling, quality assurance, record keeping and retention

Data will be managed according to the standard operating procedures of the Cancer Research UK Clinical Trials Unit (CRCTU) at the University of Birmingham, UK. The CRCTU is fully compliant with the Data Protection Act 1998 and the International Conference on Harmonisation Good Clinical Practice (ICH GCP). The CRCTU will monitor the trial and provide annual reports to the MHRA. The trial is registered with the Data Protection Act website at the University of Birmingham. Donor and patient details will be kept anonymous (specific study identification codes will be used for each study donor). Anonymised donor data will be used in future publications arising from the study. Patients will be identified using only their unique registration number, patient initials on the Case Report Form and correspondence between the Trials Office and the participating site. In addition, the patients are requested to give permission for the Trials Office to be sent a copy of their signed Informed Consent Form which will not be anonymised. This will be used to perform in-house monitoring of the consent process. Identifiable data will only be made available to authorised staff of the study sponsor, its authorised representatives and regulatory authorities. All patients will be consented specifically to enable data to be shared as detailed above. Confidentiality will otherwise be maintained throughout the trial and thereafter and data will be anonymised. On completion of the trial, data will be transferred to a secure archiving facility at the University of Birmingham, where data will be held for a minimum of 10 years and then destroyed.

For peer review only

Electronic case report forms (CRFs)

CRFs have been designed to capture as much, donor, perfusion and patient data as possible and feasible. The liver registration form and donor history form detail all that is relevant regarding the quality of the graft itself. The perfusion form enables collection of the perfusion parameters, biochemical data and the outcome of the perfusion. The patient registration and visit 1 forms will capture the demographics of the recipient as well as track the operative and post-operative course. Visits 2-4 are for patient follow-up.

Trial organisational structure

The University of Birmingham will act as single sponsor this single centre study. The trial is being conducted under the auspices of the CRCTU, The University of Birmingham according to their local procedures. The Trial Management Group (TMG) will be responsible for the day-to-day running and management of the trial. Members of the TMG include the chief investigator, co-investigators, project manager, trial management team leader, senior trial coordinator, trial coordinator, lead trial statistician, and trial statistician. The TMG will have regular meetings during recruitment. The Data Monitoring Committee (DMC) will consist of independent members Professor James Neuberger, Mr Gabi Oniscu and Professor Jacques Pirenne. Data analyses will be supplied in confidence to the independent DMC, which will be asked to give advice on whether the accumulated data from the trial, together with the results from other relevant research, justifies the continuing recruitment of further patients. The DMC will operate in accordance with a trial specific charter based upon the template created by the Damocles Group. The DMC will meet at 2 scheduled time points after the interim analyses as previously described (Figure 2). An emergency meeting may also be convened if a safety issue is identified. The DMC will report directly to both the VITTAL Trial Management Group (Chief Investigator) who will convey the findings of the DMC to the trial steering group and funders/sponsor as appropriate or when specifically requested by these parties.

Sources of funding

The VITTAL trial is funded by a grant awarded by the Wellcome Trust Health Innovation Challenge Fund (awarded December 2015).

Trial status

For peer review only

Recruitment for the trial opened in October 2016 and recruitment is expected to last 24 months.

DISCUSSION

The consequence of the escalating demand for liver transplantation is increasing waiting list mortality and in many countries, patients are more likely to die whilst waiting for an organ than in the first year after their transplant.[45] The outcomes of high-risk livers are inferior to standard grafts and the difference is most noticeable within the initial 90 days. Indeed, severe early allograft dysfunction or primary non-function often trigger post-transplant sepsis and multi-organ failure and as consequence, livers with marginal features are often declined and discarded.

Our preliminary experience and pilot transplant series showed NMP-L can provide objective information regarding liver function and the VITTAL trial aims to produce robust data and validate our initial observations.

Several challenges were identified when designing the VITTAL trial with the foremost being to create a sound definition of a discarded liver. There is an undeniable variation in utilisation of high-risk livers among the UK transplant centres which has been recognised and highlighted by NHSBT. The organisation published "Taking organ transplantation to 2020", a strategy that aims to create greater consistency in the acceptance of organ offers and utilisation of marginal livers across all centers.[46] To address this issue for this study purposes, every declined liver offered for enrolment into VITTAL has to meet also at least one of a list of predefined, constant inclusion measures, adopted in combination with a two-consultant system of macroscopic liver quality assessment.

The most important factor to consider whilst designing a trial that pushes the current boundaries of high-risk livers utilisation is patient safety. Although we opted for liberal liver graft selection inclusion criteria, only low to moderate risk recipients are eligible to take part in this trial. Such an approach has been shown previously to be the safest and the most successful strategy for utilisation of high-risk organs.[47 48] The intended recipients will be risk stratified and selected by the Liver Unit's liver transplant multi-disciplinary team. Another important trial safety feature is its three-stage adaptive design, introducing two interim safety analyses after completion of 3 and 11 transplants respectively. There are undoubtedly some

For peer review only

livers that will not be salvageable or ever safe to transplant. It is important for the purposes of the trial to include organs that fail to meet the defined viability criteria, to compare these with transplantable high-risk livers. The research work package linked with the trial was designed to identify sensitive point of care liver quality tests and propose novel biomarkers or panels associated with viable livers.

The primary end-point of 90-day patient survival has been chosen as it is a nationally accepted, monitored and continuously audited outcome following liver transplantation. Obviously, the graft survival rate is important and for the trial to truly be successful, patients who reach the primary end-point should have a VITTAL graft still *in-situ*. This will be considered when the DMC monitor the results at the interim analyses.

As well as the study design, challenges with trial logistics were also identified. One of the previously unseen difficulties after discussion with the haematology team, is the issuing of packed red cells matched to the intended recipient, potentially before the patient is admitted to hospital, to avoid delaying the start of the perfusion. When patients are listed, they undergo a blood cross-matching process to identify blood group and the presence of antibodies. This sample is not held for longer than 7 days by the hospital and so if they are admitted for a transplant or require blood products for some other intervention, they have a new sample sent before those products are issued. In the case of the VITTAL trial, a perfusion may need to commence before the patient is admitted to hospital as they may have to travel some distance. Minimising the cold ischemic time of marginal grafts is paramount to improve the chances of graft salvage. Therefore, in this scenario, blood is issued for the trial based on the results of the original sample and a repeat is sent when the patient is admitted to check they have not subsequently developed new antibodies. Blood product traceability is an important consideration and the blood products are documented to have been used in the device perfusate only and have not been used for recipient transfusion.

SUMMARY

The presented VITTAL trial is the first clinical trial designed to objectively assess function of declined livers using NMP-L and subsequently transplanting viable grafts. It is hoped that the trial will identify a proportion of discarded organs that can be successfully transplanted and the generated data will provide objective and validated information that can be subsequently implemented in the process of acceptance and allocation of high-risk donor livers. This novel

For peer review only

approach should improve consistency and increase utilisation of marginal liver grafts without compromising recipient safety.

For peer review only

ACKNOWLEDGMENTS, FINANCIAL SUPPORT AND SPONSORSHIP

This trial is funded by a grant awarded by the Wellcome Trust Health Innovation Challenge Fund (awarded in December 2015). The grant application was a joint effort of the co-applicant team (Darius Mirza, Hynek Mergental, Simon Afford, David Adams, Peter Friend, Stefan Hübscher, Julian Bion, Christina Yap) and collaborators (Thamara Perera, Hentie Cilliers, Ricky Bhogal, Richard Laing, James Ferguson, Desley Neil, Amanda Smith, Steven Youngs, Darren Barton and Leslie Russell). The project was endorsed and supported by all the clinical staff at the Liver Unit of UHBFT and we would like to thank everybody for their hard work and dedication to the transplant program.

RWL, YB and SCA have received support from the NIHR Birmingham Liver Biomedical Research Unit.

The views expressed in this publication are those of the authors and not necessarily those of the NHS, the National Institute for Health Research or the Department of Health.

AUTHORS' CONTRIBUTIONS

DFM (chief investigator) and HM (principal investigator) had the original concept of the VITTAL trial following on from a successful pilot study. DFM, HM, RWL, AK, CY and designed the VITTAL trial. RWL, MW, AK, and HM wrote the protocol and IRAS applications. DB, HM, SCA, PF and DFM reviewed all protocol versions. RWL, MW and DB (senior trials co-ordinator) submitted all REC, MHRA, HRA, NHSBT and local R&D applications. HM, AK, DFM and CY devised the statistical plan. TP, PM, JI, KR, HM and DFM are the transplant surgeons involved in the trial. RWL and MW wrote the patient information sheets, external trial information and patient CRFs. RWL, HM and DFM wrote the manuscript and all authors reviewed the final version.

CONFLICT OF INTEREST

RWL and YB receive salary as Wellcome Trust research fellows.

For peer review only

Appendix 1. Definition of adverse events

ADVERSE EVENT (AE)

Any untoward medical occurrence in a subject.

NOTE this definition does not imply that there is a relationship between the adverse event and the device under investigation.

SERIOUS ADVERSE EVENT (SAE)

An adverse event that

a) led to a death,

b) led to a serious deterioration in the health of the subject that

1) resulted in a life-threatening illness or injury,

2) resulted in a permanent impairment of a body structure or a body function,

3) required in-patient hospitalization or prolongation of existing hospitalization,

4) resulted in medical or surgical intervention to prevent permanent impairment to body structure or a body function.

c) led to foetal distress, foetal death or a congenital abnormality or birth defect.

ADVERSE DEVICE EFFECT (ADE)

Any untoward and unintended response to a medical device.

NOTE 1 This definition includes any event resulting from insufficiencies or inadequacies in the instructions for use or the deployment of the device.

SERIOUS ADVERSE DEVICE EFFECT (SADE)

Adverse device effect that has resulted in any of the consequences characteristic of a serious adverse event or that might have led to any of these consequences if suitable action had not been taken or intervention had not been made or if circumstances had been less opportune.

UNANTICIPATED SERIOUS ADVERSE DEVICE EFFECTS (USADE)

Any serious adverse device effect which, by its nature, incidence, severity or outcome, has not been identified in the anticipated AE's listed in section 8.1.1.

DEVICE DEFICIENCY

For peer review only

Inadequacy of a medical device with respect to its identity, quality, durability, reliability, safety or performance. Device deficiencies include malfunctions, use errors and inadequate labelling.

USE ERROR

Act or omission of an act that results in a different medical device response than intended by the manufacturer or expected by the user. Use error includes slips, lapses and mistakes. An unexpected physiological response of the subject does not itself constitute a use error.

SEVERITY DEFINITIONS

The following definitions will be used to determine the severity rating for all adverse events:

Mild: awareness of signs or symptoms, that does not interfere with the subject's usual activity or is transient that resolved without treatment and with no sequelae.

Moderate: a sign or symptom, which interferes with the subject's usual activity.

Severe: incapacity with inability to do work or perform usual activities.

For peer review only

Clavien Dindo Classification

Grades	*Definition*
Grade I:	Any deviation from the normal postoperative course without the need for pharmacological treatment or surgical, endoscopic and radiological interventions. This grade also includes wound infections opened at the bedside.<br>[Allowed therapeutic regimens are: drugs as anti-emetics, antipyretics, analgesics, diuretics and electrolytes and physiotherapy.]
Grade II:	Requiring pharmacological treatment with drugs (other than those noted above) including blood transfusions and total parenteral nutrition
Grade III:	Requiring surgical, endoscopic or radiological intervention
Grade III-a:	intervention not under general anaesthesia
Grade III-b:	intervention under general anaesthesia
Grade IV:	Life-threatening complication (including CNS complications)[1] requiring HDU/ICU management
Grade IV-a:	single organ dysfunction (includes dialysis)
Grade IV-b:	multi organ dysfunction
Grade V:	Death of a patient
Suffix 'd':	If the patients suffers from a complication at the time of discharge, the suffix “d” (for ‘disability’) is added to the respective grade of complication.

[1]brain haemorrhage, ischaemic stroke, subarachnoid haemorrhage, but excluding transient ischaemic attacks; HDU, high dependency unit; ICU, intensive care unit.

For peer review only

References

1. Williams R, Aspinall R, Bellis M, et al. Addressing liver disease in the UK: a blueprint for attaining excellence in health care and reducing premature mortality from lifestyle issues of excess consumption of alcohol, obesity, and viral hepatitis. *Lancet* 2014;384(9958):1953-97. doi: 10.1016/S0140-6736(14)61838-9

2. Williams R, Ashton K, Aspinall R, et al. Implementation of the Lancet Standing Commission on Liver Disease in the UK. *Lancet* 2015;386(10008):2098-111. doi: 10.1016/S0140-6736(15)00680-7

3. Mathur AK, Ranney DN, Patel SP, et al. The effect of smoking on biliary complications following liver transplantation. *Transpl Int* 2011;24(1):58-66. doi: 10.1111/j.1432-2277.2010.01146.x [published Online First: 2010/08/26]

4. NHS Blood and Transplant. Organ Donation and Transplantation Activity Report 2015/16, 2016.

5. NHS Blood and Transplant. Annual report on liver transplanation 2015/16, 2016.

6. Sotiropoulos GC, Radtke A, Molmenti EP, et al. Long-term follow-up after right hepatectomy for adult living donation and attitudes toward the procedure. *Ann Surg* 2011;254(5):694-700; discussion 00-1. doi: 10.1097/SLA.0b013e31823594ae

7. Simoes P, Kesar V, Ahmad J. Spectrum of biliary complications following live donor liver transplantation. *World J Hepatol* 2015;7(14):1856-65. doi: 10.4254/wjh.v7.i14.1856

8. Mirza DF, Gunson BK, Da Silva RF, et al. Policies in Europe on "marginal quality" donor livers. *Lancet* 1994;344(8935):1480-3.

9. NHS Blood and Transplant. Organ Donation and Transplantation Activity Report 2014/15, 2015.

10. Working Party of The British Transplantation Society. Transplantation from donors after deceased circulatory death, 2013.

11. Feng S, Goodrich NP, Bragg-Gresham JL, et al. Characteristics associated with liver graft failure: the concept of a donor risk index. *Am J Transplant* 2006;6(4):783-90.

12. Braat AE, Blok JJ, Putter H, et al. The Eurotransplant donor risk index in liver transplantation: ET-DRI. *Am J Transplant* 2012;12(10):2789-96. doi: 10.1111/j.1600-6143.2012.04195.x

13. Belzer FO, Southard JH. Principles of solid-organ preservation by cold storage. *Transplantation* 1988;45(4):673-6. [published Online First: 1988/04/01]

14. Carini R, Autelli R, Bellomo G, et al. Alterations of cell volume regulation in the development of hepatocyte necrosis. *Exp Cell Res* 1999;248(1):280-93. doi: 10.1006/excr.1999.4408

For peer review only

15. Pratschke J, Weiss S, Neuhaus P, et al. Review of nonimmunological causes for deteriorated graft function and graft loss after transplantation. *Transpl Int* 2008;21(6):512-22. doi: 10.1111/j.1432-2277.2008.00643.x

16. Menger MD, Pelikan S, Steiner D, et al. Microvascular ischemia-reperfusion injury in striated muscle: significance of "reflow paradox". *Am J Physiol* 1992;263(6 Pt 2):H1901-6.

17. Vogel T, Brockmann JG, Coussios C, et al. The role of normothermic extracorporeal perfusion in minimizing ischemia reperfusion injury. *Transplant Rev (Orlando)* 2012;26(2):156-62. doi: 10.1016/j.trre.2011.02.004

18. Henry SD, van der Wegen P, Metselaar HJ, et al. Hydroxyethyl starch-based preservation solutions enhance gene therapy vector delivery under hypothermic conditions. *Liver Transpl* 2008;14(12):1708-17. doi: 10.1002/lt.21623

19. Mediavilla MG, Krapp A, Carrillo N, et al. Efficient cold transfection of pea ferredoxin-NADP(H) oxidoreductase into rat hepatocytes. *J Gene Med* 2006;8(3):306-13. doi: 10.1002/jgm.847

20. Ke B, Shen XD, Tsuchihashi S, et al. Viral interleukin-10 gene transfer prevents liver ischemia-reperfusion injury: Toll-like receptor-4 and heme oxygenase-1 signaling in innate and adaptive immunity. *Hum Gene Ther* 2007;18(4):355-66. doi: 10.1089/hum.2007.181

21. Le Moine O, Louis H, Demols A, et al. Cold liver ischemia-reperfusion injury critically depends on liver T cells and is improved by donor pretreatment with interleukin 10 in mice. *Hepatology* 2000;31(6):1266-74. doi: 10.1053/jhep.2000.7881

22. Ambiru S, Uryuhara K, Talpe S, et al. Improved survival of orthotopic liver allograft in swine by addition of trophic factors to University of Wisconsin solution. *Transplantation* 2004;77(2):302-19. doi: 10.1097/01.TP.0000100468.94126.AF

23. Koti RS, Seifalian AM, Davidson BR. Protection of the liver by ischemic preconditioning: a review of mechanisms and clinical applications. *Dig Surg* 2003;20(5):383-96. doi: 72064

24. Raeburn CD, Zimmerman MA, Arya J, et al. Ischemic preconditioning: fact or fantasy? *J Card Surg* 2002;17(6):536-42.

25. Oniscu GC, Randle LV, Muiesan P, et al. In situ normothermic regional perfusion for controlled donation after circulatory death-the United kingdom experience. *Am J Transplant* 2014;14(12):2846-54. doi: 10.1111/ajt.12927

26. Ravikumar R, Jassem W, Mergental H, et al. Liver transplantation after ex vivo normothermic machine preservation: a Phase 1 (first-in-man) clinical trial. *Am J Transplant* 2016 doi: 10.1111/ajt.13708

27. Tuttle-Newhall JE, Collins BH, Kuo PC, et al. Organ donation and treatment of the multi-organ donor. *Curr Probl Surg* 2003;40(5):266-310. doi: 10.1016/S0011-3840(03)00011-X

For peer review only

28. Frost AE. Donor criteria and evaluation. *Clin Chest Med* 1997;18(2):231-7.

29. Pirenne J, Monbaliu D, Van Gelder F, et al. Liver transplantation using livers from septuagenarian and octogenarian donors: an underused strategy to reduce mortality on the waiting list. *Transplant Proc* 2005;37(2):1180-1. doi: 10.1016/j.transproceed.2004.12.168

30. Biancofiore G, Bindi M, Ghinolfi D, et al. Octogenarian donors in liver transplantation grant an equivalent perioperative course than ideal young donors. *Dig Liver Dis* 2017 doi: 10.1016/j.dld.2017.01.149

31. Ferla F, De Carlis R, Mariani A, et al. Liver transplant using octogenarian donors. *Liver Transpl* 2016;22(7):1040-1. doi: 10.1002/lt.24450

32. Hellinger A, Fiegen R, Lange R, et al. Preservation of pig liver allografts after warm ischemia: normothermic perfusion versus cold storage. *Langenbecks Archiv fur Chirurgie* 1997;382(4):175-84.

33. Schon MR, Kollmar O, Wolf S, et al. Liver transplantation after organ preservation with normothermic extracorporeal perfusion. *Ann Surg* 2001;233(1):114-23.

34. Imber CJ, St Peter SD, Lopez de Cenarruzabeitia I, et al. Advantages of normothermic perfusion over cold storage in liver preservation. *Transplantation* 2002;73(5):701-9. [published Online First: 2002/03/22]

35. Butler AJ, Rees MA, Wight DG, et al. Successful extracorporeal porcine liver perfusion for 72 hr. *Transplantation* 2002;73(8):1212-8.

36. Brockmann J, Reddy S, Coussios C, et al. Normothermic perfusion: a new paradigm for organ preservation. *Ann Surg* 2009;250(1):1-6. doi: 10.1097/SLA.0b013e3181a63c10

37. Perera M, Mergental H, Stephenson B, et al. First human liver transplantation using a marginal allograft resuscitated by normothermic machine perfusion. *Liver Transplant* 2015 doi: 10.1002/lt.24369

38. Simon R. Optimal two-stage designs for phase II clinical trials. *Control Clin Trials* 1989;10(1):1-10.

39. Chen TT. Optimal three-stage designs for phase II cancer clinical trials. *Stat Med* 1997;16(23):2701-11.

40. Dindo D, Demartines N, Clavien PA. Classification of surgical complications: a new proposal with evaluation in a cohort of 6336 patients and results of a survey. *Ann Surg* 2004;240(2):205-13. [published Online First: 2004/07/27]

41. Dutkowski P, Oberkofler CE, Slankamenac K, et al. Are there better guidelines for allocation in liver transplantation? A novel score targeting justice and utility in the model for end-stage liver disease era. *Ann Surg* 2011;254(5):745-53; discussion 53. doi: 10.1097/SLA.0b013e3182365081

42. NHSBT. Activity report 2014/15, 2014/15.

For peer review only

43. Wilson EB. Probable Inference, the Law of Succession, and Statistical Inference. . *JASA* 1927;22(158):209-12.

44. Dutkowski P, Schlegel A, Slankamenac K, et al. The use of fatty liver grafts in modern allocation systems: risk assessment by the balance of risk (BAR) score. *Ann Surg* 2012;256(5):861-8; discussion 68-9. doi: 10.1097/SLA.0b013e318272dea2

45. NHSBT, NHSEngland. Annual Report on Liver Transplantation: Report for 2015/2016. September 2016 [published Online First: Sept 2016]

46. Walker KG, Eastmond CJ, Best PV, et al. Eosinophilia-myalgia syndrome associated with prescribed L-tryptophan. *Lancet* 1990;336(8716):695-6.

47. Avolio AW, Halldorson JB, Burra P, et al. Balancing utility and need by means of donor-to-recipient matching: a challenging problem. *Am J Transplant* 2013;13(2):522-3. doi: 10.1111/ajt.12031

48. Dutkowski P, Oberkofler CE, Slankamenac K, et al. Are there better guidelines for allocation in liver transplantation? A novel score targeting justice and utility in the model for end-stage liver disease era. *Ann Surg* 2011;254(5):745-53; discussion 53. doi: 10.1097/SLA.0b013e3182365081 [published Online First: 2011/11/02]

For peer review only

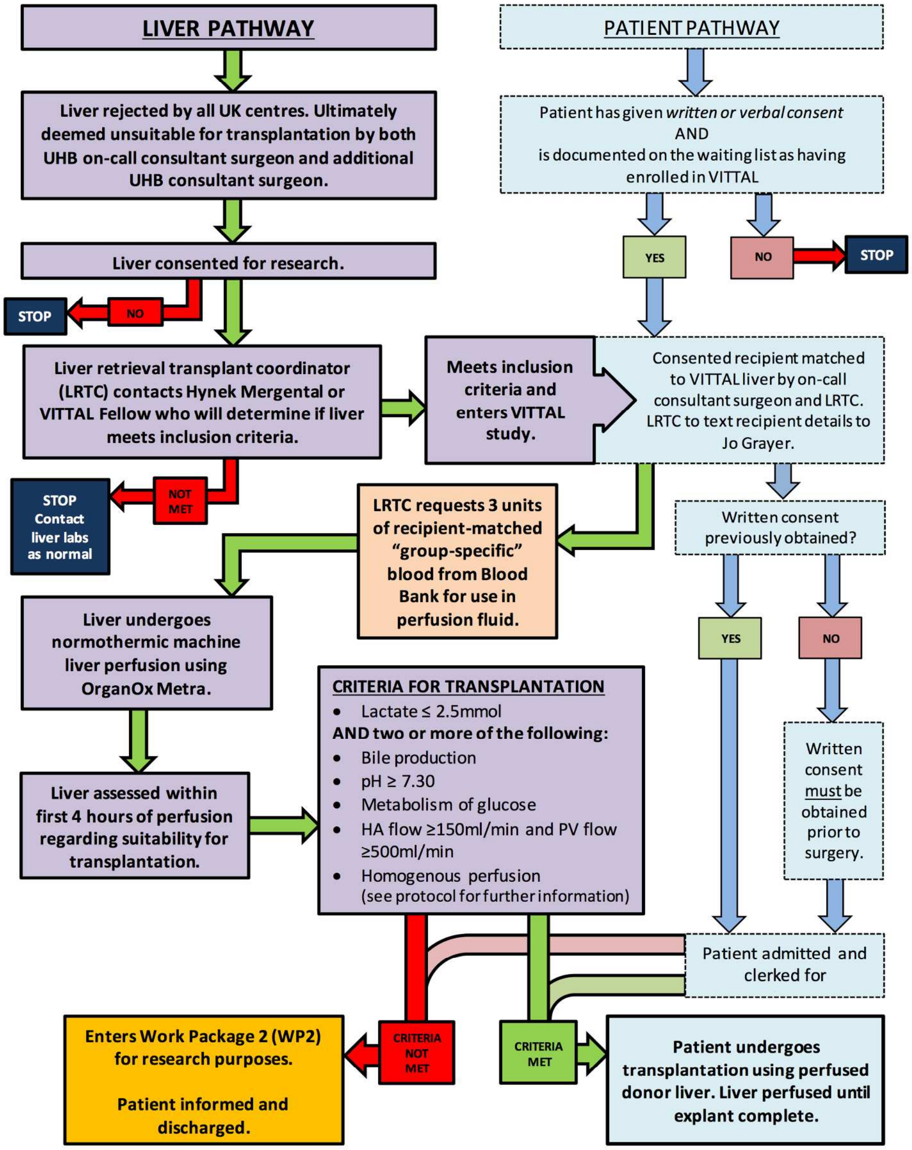

Figure 1. Patient and donor liver pathways

179x230mm (300 x 300 DPI)

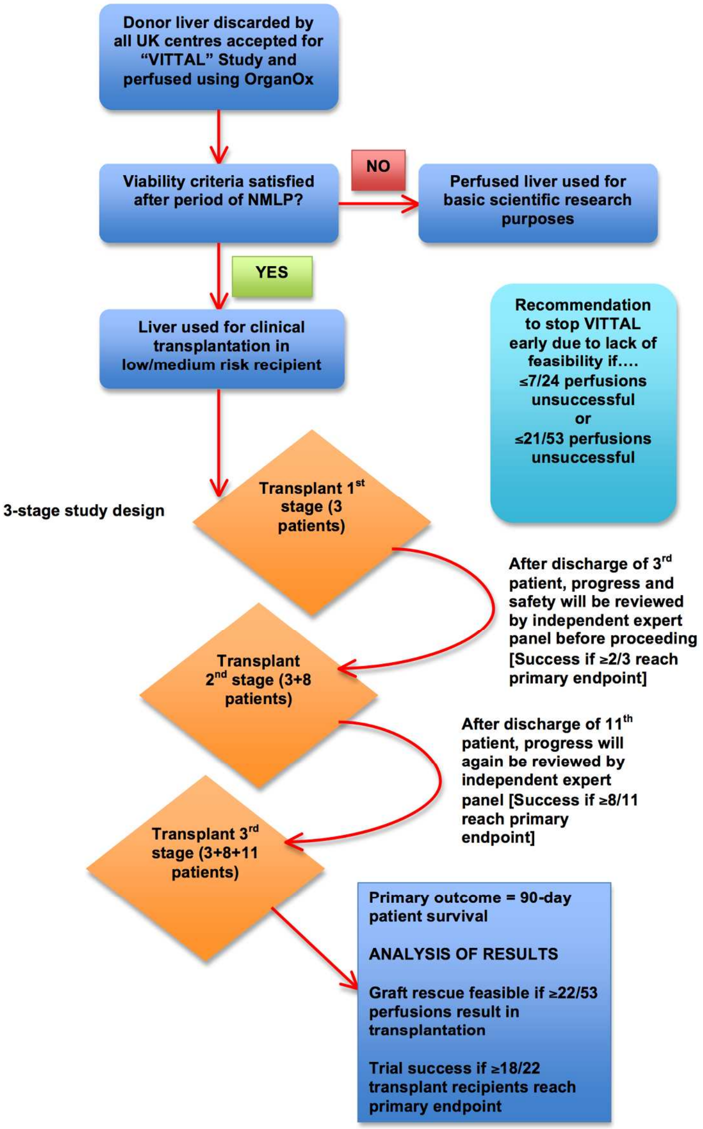

Figure 2. Trial schema

145x217mm (300 x 300 DPI)

BMJ Open

Viability testing and transplantation of marginal livers (VITTAL) using normothermic machine perfusion: A study protocol for an open label, non-randomised, prospective, single arm trial

Journal:	*BMJ Open*
Manuscript ID	bmjopen-2017-017733.R1
Article Type:	Protocol
Date Submitted by the Author:	26-Jun-2017
Complete List of Authors:	Laing, Richard; University of Birmingham, National Institute for Health Research (NIHR) Birmingham Liver Biomedical Research Unit; University Hospitals Birmingham NHS Foundation Trust, Liver Unit<br>Mergental, Hynek; University Hospitals Birmingham NHS Foundation Trust, Liver Unit<br>Yap, Christina; University of Birmingham, NIHR Liver BRU Clinical trials group (EDD), CRUK clinical trials unit<br>Kirkham, Amanda; University of Birmingham, NIHR Liver BRU Clinical trials group (EDD), CRUK clinical trials unit<br>Wilkhu, Manpreet; University of Birmingham, NIHR Liver BRU Clinical trials group (EDD), CRUK clinical trials unit<br>Barton, Darren; University of Birmingham, NIHR Liver BRU Clinical trials group (EDD), CRUK clinical trials unit<br>Curbishley, Stuart; University of Birmingham, National Institute for Health Research (NIHR) Birmingham Liver Biomedical Research Unit<br>Boteon, Yuri; University of Birmingham, National Institute for Health Research (NIHR) Birmingham Liver Biomedical Research Unit; University Hospitals Birmingham NHS Foundation Trust, Liver Unit<br>Neil, Desley; University Hospitals Birmingham NHS Foundation Trust, Histopathology<br>Hubscher, Stefan; University Hospitals Birmingham NHS Foundation Trust, Histopathology<br>Perera, T; University Hospitals Birmingham NHS Foundation Trust, Liver Unit<br>Muiesan, Paolo; University Hospitals Birmingham NHS Foundation Trust, Liver Unit<br>Isaac, John; University Hospitals Birmingham NHS Foundation Trust, Liver Unit<br>Roberts, Keith; University Hospitals Birmingham NHS Foundation Trust, Liver Unit<br>Cilliers, Hentie; University Hospitals Birmingham NHS Foundation Trust, Liver Unit<br>Afford, Simon; University of Birmingham, National Institute for Health Research (NIHR) Birmingham Liver Biomedical Research Unit<br>Mirza, Darius; University Hospitals Birmingham NHS Foundation Trust, Liver Unit
**Primary Subject Heading**:	Surgery

For peer review only - http://bmjopen.bmj.com/site/about/guidelines.xhtml

Secondary Subject Heading:	Gastroenterology and hepatology
Keywords:	Hepatobiliary disease < GASTROENTEROLOGY, Hepatology < INTERNAL MEDICINE, Hepatobiliary tumours < ONCOLOGY, Transplant surgery < SURGERY, TRANSPLANT SURGERY

SCHOLARONE™ Manuscripts

For peer review only

Viability testing and transplantation of marginal livers (VITTAL) using normothermic machine perfusion: Study protocol for an open label, non-randomised, prospective, single arm trial

Richard W. Laing[1,2*], Hynek Mergental[1,2*], Christina Yap[3], Amanda Kirkham[3], Manpreet Whilku[3], Darren Barton[3], Stuart Curbishley[2], Yuri L. Boteon[1,2], Desley A. Neil[1], Stefan G. Hübscher[1,2], M. Thamara P. R. Perera[1,2], Paolo Muiesan[1,2], John Isaac[1], Keith J. Roberts[1], Hentie Cilliers[1], Simon C. Afford[2], Darius F. Mirza[1,2].

1. Liver Unit, Queen Elizabeth Hospital, University Hospitals Birmingham NHS Foundation Trust, UK
2. National Institute for Health Research (NIHR) Birmingham Liver Biomedical Research Unit, Institute of Immunology and Immunotherapy, University of Birmingham, UK
3. Cancer Research UK Clinical Trials Unit, Institute of Cancer and Genomic Sciences, University of Birmingham, UK

ADDRESS FOR CORRESPONDENCE
Professor Darius F Mirza, FRCS
Liver Unit, Queen Elizabeth Hospital Birmingham
University Hospitals Birmingham NHS Foundation Trust
Birmingham, B15 2TH
United Kingdom
Email: Darius.Mirza@uhb.nhs.uk
Tel: 0044 0121 371 4638
Fax: 0044 0121 414 1833

Word Count 7729

For peer review only

LIST OF ABBREVIATIONS

ALT, alanine transaminase;

AST, aspartate transaminase;

BAR, balance of risk score;

CIT, cold ischemic time;

CRCTU, Cancer Research UK Clinical Trials Unit;

DBD, donation after brain-stem death;

DCD, donation after circulatory death;

DRI, donor risk index;

eCRF, electronic case report form;

FWIT, functional warm ischemic time;

HMP, hypothermic machine perfusion;

HRA, Health Research Authority;

ICU, intensive care unit;

MHRA, Medicines and Healthcare Products Regulatory Agency;

MRCP, magnetic resonance imaging of the biliary and pancreatic tree;

NHSBT, National Health Service Blood and Transplant;

NMP, normothermic machine perfusion;

NMP-L, normothermic machine perfusion of the liver;

RINTAG, Research, Innovation and Novel Technologies Advisory Group;

SCS, static cold storage;

UHBFT, University Hospitals Birmingham NHS Foundation Trust;

VITTAL, Viability testing and transplantation of marginal livers.

For peer review only

ABSTRACT

Introduction

The use of marginal, or extended criteria donor livers is increasing. These organs carry a greater risk of initial dysfunction and early failure, as well as inferior long-term outcomes. As such, many are rejected due to a perceived risk of use and utilisation varies widely between centres. Ex-situ normothermic machine perfusion of the liver (NMP-L) may enable the safe transplantation of organs that meet defined objective criteria denoting their high-risk status and are currently being declined for use by all the UK transplant centres.

Methods and analysis

VITTAL (Viability testing and transplantation of marginal livers) is an open label, non-randomised, prospective, single arm trial designed to determine whether currently unused donor livers can be salvaged and safely transplanted with equivalent outcomes in terms of patient survival. The procured rejected livers must meet pre-defined criteria that objectively denote their marginal condition. The liver is subjected to NMP-L following a period of static cold storage. Organs metabolising lactate to ≤2.5mmol/L within 4 hours of the perfusion commencing in combination with two or more of the following parameters – bile production, metabolism of glucose, a hepatic arterial flow rate ≥150ml/min and a portal venous flow rate ≥500ml/min, a pH ≥7.30 and/or maintain a homogenous perfusion – will be considered viable and transplanted into a suitable consented recipient. The co-primary outcome measures are the success rate of NMP-L to produce a transplantable organ and 90-day patient post-transplant survival.

Ethics and dissemination

The protocol was approved by the National Research Ethics Service (London – Dulwich Research Ethics Committee, 16/LO/1056), the Medicines and Healthcare Products Regulatory Agency and is endorsed by the National Health Service Blood and Transplant Research, Innovation and Novel Technologies Advisory Group. The findings of this trial will be disseminated through national and international presentations and peer-reviewed publications.

Registration

Clinicaltrials.gov NCT02740608

Strengths and Limitations of the Study

For peer review only

- The study will answer the question: "Can ex-situ end-ischemic normothermic machine perfusion safely increase the number of transplantable livers?"
- The study aims to establish objective liver viability criteria and biomarkers that may enable point-of-care assessment of liver quality
- The study has clearly defined criteria characterising the discarded organs
- Incorporation of an adaptive three-stage trial design provides opportunities to assess patients' safety, allowing for early trial termination if necessary
- The trial includes low and moderate risk recipients only – the suitability for high-risk recipients will require further testing

Keywords

Liver transplant, extended criteria donor, machine perfusion, normothermic, viability testing.

For peer review only

INTRODUCTION

Liver transplantation

Liver transplantation is a highly successful treatment for end stage liver disease, fulminant hepatic failure and early stage primary liver cancer. Deaths from liver disease have soared by 40 per cent in a decade and continue to rise. Liver disease kills 11,000 a year in England and the average age of death from liver disease (59 years), continues to decrease.[1] Over the past 50 years, transplant techniques and outcomes have greatly improved and 5-year survival rates of 70-80% mean that transplantation has become the mainstay of treatment for an increasing number of patients with chronic liver disease, metabolic disorders, acute liver failure and malignancy. As such, the demand for donor livers greatly exceeds supply and approximately 20% of patients die whilst awaiting transplantation.[2] In Europe, the most common indications for liver transplantation are cirrhosis (68%), malignancy (14%), and acute hepatic failure (8%). The main causes for cirrhosis in Europe are the hepatotropic viruses and alcohol related liver disease.[3] Non-alcoholic fatty liver disease is an emergent cause and despite health campaigns, the incidence continues to rise. In the UK, it is predicted that the incidences of end stage liver disease and hepatocellular carcinoma will increase substantially during the next decade, exacerbating the existing shortage of donor livers.

The UK Liver Transplant Programme

Between March 2015 and April 2016, there were 1161 new waiting list registrations in the UK, and 878 transplants were carried out. Of the 621 patients on the list as of April 2015, 22% died or were removed from the list (n=135) due to deteriorating health.[4] This is reflected across other countries to the extent that a patient is now more likely to die within the first 12 months of being listed than the first 12 months' post-transplant.[5] Over the past decade there has been a very modest increase in the use of standard or 'ideal' organ donors (those retrieved from young donors following a diagnosis of brain-stem death, DBD). In response, centres have utilised donors following circulatory death (DCD) and sub-optimal "marginal" or "extended criteria" donors (those of older age, livers with a presence of steatosis etc.).

Responding to the shortage

There are several ways to respond to the shortage. Organ donation policies are undergoing changes however there is a lack of well-controlled scientific evidence on which to base decisions regarding policy-making and opinions are strong and divided. Spain has the highest organ donation rates and operates an opt-out system, however the rise in rates only started

For peer review only

approximately 10 years after the system's introduction. Wales is the most recent country to go down this route, however unlike in Spain, next of kin consent is still required before patients can become organ donors. More likely, the increased Spanish donation rates are due to a combination of factors – the creation of a transplant coordination network that operates at hospital, regional and national levels, the placement of transplant coordinators at each procurement hospital and the improvement in the quality of information received by the public. Living donation is one potential means to increase the number of liver transplants, using surgical techniques developed for liver resection and 'liver splitting' (which uses a single liver for transplantation into two recipients). The major limitations are most patients do not have a willing or suitable living donor and there are concerns about the risks to the healthy donor. The reported risk of donor death is estimated at 0.2% but the risk of serious complications is much higher.[6 7] Although programmes have had some success in countries without deceased donor programmes, living donor transplantation will be unlikely to have a significant impact on the shortage of donor livers in most countries.

The use of "marginal" or "extended criteria" donors

As discussed, a rising proportion of transplants are carried out using "marginal" or "extended criteria" grafts, procured from obese or elderly donors with multiple co-morbidities.[8] These livers are significantly more susceptible to cold storage-related ischaemic injury, which increases the risk of graft failure, and recipient morbidity and mortality. Reflecting the issues with these sub-optimal grafts, in 2014/15, of 1282 solid organ donors, only 924 (72.1%) livers were deemed suitable for retrieval and only 812 (63.3%) were subsequently transplanted.[9] The duration of the functional warm ischemic time (FWIT) is an important determinant of outcome. The recent document 'Donation After Circulatory Death' published by a steering group on behalf of the British Transplantation Society and Intensive Care Society suggested that the stand-down time from the onset of functional warm ischaemia for DCD liver transplantation was 30 minutes (although 20 minutes is ideal), and that age was an important factor. Because of this, a number of livers will be retrieved from DCDs that fall into the "marginal donor" category and may not go on to be transplanted.[10]

Several donor parameters have been identified as relative risk factors for poor outcome including age; steatosis; DCD donation; split livers; prolonged cold ischaemia time (>12 hours). These were all developed using North American data and formulated into an algorithm known as the Donor Risk Index (DRI), and later validated using European data.[11 12]

For peer review only - http://bmjopen.bmj.com/site/about/guidelines.xhtml

The British Transplantation Society have published their own guidelines on the utilisation of donor organs and use criteria in Table 1 to distinguish between grafts of varying quality.

Table 1. Criteria for donor quality as per British Transplantation Society UK Guidelines for Donors after Circulatory death

Good livers – *All should be used* (DBDs and DCDs)	Ideal Livers – *All should be used* (DCDs)	Marginal Donors – *Use selectively* (DCDs)	Absolute contraindications to using liver as donor organ
• Age <50<br>• Normal LFTs<br>• <5 days on ICU<br>• Low levels of inotropic support<br>• <30% Steatosis<br>• No active sepsis	• Age <50 years<br>• Weight <100kg<br>• FWIT <20 mins<br>• CIT <8 hours<br>• <15% Steatosis<br>• ICU stay <5 days	• Age >50 years<br>• Weight >100kg<br>• FWIT 20-30mins<br>• CIT 8-12 hrs<br>• >15% Steatosis<br>• ICU stay >5 days	• DCD with macrosteatosis steatosis >30%<br>• ESLD<br>• Acute liver failure<br>• Acute liver injury that's not improving

CIT, cold ischemic time; DBD, donor following brain death; DCD, donor following circulatory death; ESLD, end-stage liver disease; FWIT, functional warm ischemic time; ICU, intensive care stay; LFTs, liver function tests

Organ Preservation

The current standard of donor liver preservation is based on static cold storage (SCS).[13] During SCS, organs are flushed and cooled with specific chilled preservation solutions (University of Wisconsin [UW] solution is used most commonly although Histidine-Tryptophan-Ketoglutarate (HTK) solution is also used less widely) and ice is added to the abdominal cavity. After retrieval, the organ is placed in fluid-filled sterile plastic bags for transportation and stored in preservation solution within an ice-box until transplantation. Although the available preservation solutions differ in chemical composition, their function is essentially the same. The hypothermia aims to reduce the liver's metabolic activity and the solution aims to reduce the cellular swelling. This is a consequence of anaerobic metabolism resulting in depletion of adenosine triphosphate stores leading to influx of free calcium and activation of phospholipases.[14] Cooling the organ slows metabolism approximately 12-fold but cannot prevent its dysfunction and the eventual destruction of cellular integrity. Ischaemia-reperfusion is an important factor influencing graft outcome.[15] The ischaemic phase starts early in the procurement process (swings in blood pressure following brain-death or due to the functional warm ischaemic time in non-heart beating donors) and triggers a complex cascade of cellular and molecular events including the release of pro-inflammatory

For peer review only

mediators and chemotaxis of cell types that initiate progressive immunological processes. During the reperfusion phase, "the reflow paradox" causes infiltration of the tissues by leucocytes and cellular injury occurs through a series of pathways that include lipid peroxidation and the creation of reactive oxygen species[16] The most common manifestation of the ischaemia-reperfusion process is delayed graft function, which is the inability of the organ to fulfil the physiological needs of the recipient and is associated with graft failure, re-transplantation and death.[17] Static cold storage therefore is unable to reverse the injury sustained during donor death and procurement, causes injury due to the cooling process, limits the preservation time and prevents physiological assessment prior to transplantation.

In-situ organ reconditioning

To reverse or diminish the injury, many cytoprotective strategies have been tested in experimental models of transplantation and several have been shown to have therapeutic potential, including gene therapy,[18] [19] cytokine or growth factor administration,[20-22] vasodilating agents and ischemic pre-conditioning.[23] [24] Treatment of the organ during preservation has major logistic and ethical advantages over any attempt to achieve the same effects by treating the donor (therapeutic interventions before declaration of death are not currently permitted unless they are of potential benefit to the donor). Recently there has been published early experience with normothermic regional perfusion of DCD donors, nevertheless the feasibility and benefit of this experimental approach is yet to be shown.[25]

Normothermic Machine Perfusion of the Liver (NMP-L)

Bretschneider and Starzl first attempted machine perfusion of the liver in the late 1960's. Although hypothermic machine perfusion (HMP) has been shown some promise in clinical studies, NMP-L combats the limitations of SCS previously described by aiming to maintain the organ at the body's natural temperature while providing oxygen, nutrition and the essential substrates necessary for adequate cellular metabolism. Providing a homeostatic environment theoretically enables us to extend our storage period and test the organs physiological parameters. To date only one clinical trial of 20 adult recipients of livers maintained by HMP has been published showing a reduction in early graft dysfunction (5% vs 25% $p<0.08$) as well as a significant reduction in serum injury markers in the HMP group. A joint pilot trial between Oxford University, King's College Hospital London and University Hospitals Birmingham Foundation Trust (UHBFT) recruited 20 patients into a NMP-L phase 1 study and concluded the procedure was feasible and safe when used on

For peer review only

current conventional donor acceptance criteria.[26] Following this, a 220-patient phase III international clinical trial entitled "COPE WP2" has completed recruitment and the results are eagerly awaited. The Liver Unit at University Hospital Birmingham NHS Foundation Trust contributed to this multi-centre international trial by randomising 50% of the study patients.

Our group believes NMP-L enables the donor organ to be functionally assessed, thereby increasing transplant safety. It can also extend organ preservation times to improve transplant logistics and donor organ utilisation. There are several devices available on the market, but only the OrganOx *metra* ™ has been widely used in the clinical transplant setting.[26] Our team has performed over 70 liver transplants with grafts preserved on this machine and has gained broad experience by using this device. The OrganOx *metra* ™ is the leading device in terms of the number of clinical transplants undertaken, with more than 100 machine-perfused livers transplanted in the Phase III randomised European trial, together with 20 livers in the Phase I safety study and further on-going trials in North America. For these reasons, we have decided to use the OrganOx *metra* ™ device for the proposed study.

The device consists of a unit that cradles the liver, a perfusate reservoir, oxygenators, pumps operating at physiological pressures and a closed tubing system that connects the unit to the portal vein, hepatic artery and vena cava. The constituents of the perfusate can vary but generally consist of whole blood for oxygen carriage, sources of nutrition (glucose, insulin, amino acids), anti-thrombotic agents (heparin, epoprostenol), antibiotics and acid-base agents which help reduce cellular oedema, cholestasis, microvascular injury and the effects of free-radicals.

Benefits of NMP-L

NMP-L does not simply benefit marginal DCD organs that have been exposed to a damaging FWIT. Brain-stem death is a catastrophic physiological event associated with profound hypotension (parasympathetic response) followed by hypertension, tachycardia and high levels of circulating catecholamines (sympathetic surge) followed by another reduction in the sympathetic outflow. These dramatic swings can cause significant graft ischaemia prior to retrieval. Diabetes insipidus occurs in 70-80% of brain dead patients causing severe hypernatremia (associated with primary liver graft non-function), hypokalaemia, hypocalcaemia, hypophosphatemia and hypomagnesaemia.[27 28] Pirenne *et* al described seven

For peer review only

cases when livers from DBD's between 70 and 80 years old were used with "favourable outcomes".[29] More recently, groups from Italy have reported excellent outcomes using grafts from octogenarian donors.[30] [31] NMP-L could however play an important role in preconditioning and assessing such organs prior to transplantation.

Hellinger et al were unable to identify a benefit using NMP-L in 1997; however, it was the first study of its kind.[32] In 2001, Schon used NMP-L to preserve and re-condition livers that had been exposed to 1 hour of warm ischaemia. These livers were then transplanted into pigs which all survived longer than 7 days. The group that received livers preserved using SCS had no survivors.[33] Several studies have been published by the Oxford group, responsible for OrganOx *metra*™. Imber *et al* published results from a study on a porcine model comparing NMP-L with SCS controls. They showed livers preserved using NMP-L were significantly superior ($P<0.05$) to SCS livers "in terms of bile production, factor V production, glucose metabolism, and galactose clearance", whilst SCS livers had higher perfusate levels of hepatocellular enzymes and more cellular damage.[34] The same year they successfully perfused and maintained 5 porcine livers for 72 hours, managing to maintain normal physiological parameters, pH, protein synthesis and histological architecture.[35] In 2009, Brockman et al simulated DBD and DCD scenarios in a porcine model. After five hours of preservation (NMP-L vs SCS) there was no difference seen in preservation method in either the DCD or DBD graft recipients. After 20 hours of preservation however, both DCD and DBD grafts that had been preserved using NMP-L were superior to their SCS counterparts with respect to enzyme release, histological changes and recipient survival. Of note, there was no difference in survival between DCD and DBD NMP-L-preserved graft recipients (83% and 86% respectively).[36]

Pre-clinical research and pilot study

Our team's pre-clinical research on rejected human livers has demonstrated that metabolism of lactate, in combination with bile production, maintenance of physiological pH, and stable blood flow rates, are sensitive parameters predictive of organ viability. In April 2014, the UHBFT Novel Therapeutics Committee approved a pilot clinical project for transplantation of five reconditioned liver grafts, initially deemed unusable for transplantation. In this series, livers were declined by all the UK transplant units, after which NMP-L commenced following a variable period of SCS. Still, five out of six tested livers met the viability criteria and were successfully transplanted.[37] Although this pilot project showed that viability testing

For peer review only

has the potential to transform the organ selection and acceptance process of high-risk livers, our observation primarily provided the feasibility and short-term outcome data. In addition, this cohort also demonstrated the feasibility of performing NMP-L within a "back-to-base" model, i.e. following SCS and inspection at the transplant centre. This offers logistical and financial advantages over using NMP-L in place of SCS and may target livers that would benefit the most from NMP-L. More research in this area is required and this was recognised by the Health Innovation Challenge Committee of the Wellcome Trust who awarded our study group a research grant to fund this trial. We have demonstrated so far, that a proportion of currently rejected liver allografts might be salvaged by subjecting them to NMP-L and viability testing. Use of this technology could transform the utilisation of high-risk organs and may improve access to treatment for thousands of patients awaiting liver transplantation globally.

METHODS

Study Design Overview

VITTAL is an open label, non-randomised, prospective, single arm trial, using normothermic machine liver perfusion (NMP-L) testing viability and transplantation of marginal livers. It is being conducted at a single site (UHBFT). The design utilises two linked components assessing: (A) - the feasibility of NMP-L as a technique to increase the number of transplantable livers; and (B) - achievement of successful transplantation of the NMP-L treated marginal livers. (A) utilises a two-stage adaptive design[38], requiring up to 53 marginal livers to be perfused. (B) utilises a three-stage adaptive design[39] and requires 22 NMP-L treated marginal livers to be transplanted. Success is measured by 90-day patient survival – a nationally accepted, monitored and continually audited outcome following liver transplantation.

Ethical and regulatory approval

The National Research Ethics Service (NRES) London-Dulwich (REC reference 16/LO/1056, Protocol number RG 15-240) and the Medicines and Healthcare Products Regulatory Agency (MHRA) approved all versions of the study protocol. This trial will use the OrganOx *metra* ™ device following a variable period of SCS to evaluate organ viability pre-transplant procedure. The OrganOx *metra* ™ device currently has a CE mark for liver organ transport and not organ evaluation. The use of the device within this clinical trial is therefore off registration and UK Competent authority (MHRA) clinical trial Investigation:

For peer review only - http://bmjopen.bmj.com/site/about/guidelines.xhtml

No Objection was obtained (MHRA ref: CI/2016/0031. In addition, approval from the Research and Development (R&D) department at UHBFT and from NHSBT's RINTAG was obtained prior to the start of screening.

Graft entry into study and subsequent preparation

The patient and donor liver pathways can be seen in Figure 1. All livers will be retrieved with the intention and standardised technique to use them for transplantation. Following the retrieval procedure at the donor hospital the liver will be placed in ice-cold preservation solution on the back-table and transported (according to local protocol). If the liver is allocated to UHBFT, if it is then considered not suitable for use it must be rejected by the on-call transplanting surgeon. For the liver to be considered un-transplantable, the liver will be inspected by the on-call transplant surgeon and another transplant surgeon in the department. The liver will then be offered as a Fast Track graft to the other centres around the UK. If rejected by all centres and if consent for research was taken, it will be considered for use in VITTAL. Livers offered to our unit as Fast Track offers from other centres will undergo the same 2-consultant rejection process. An appropriate consented potential recipient will be selected by the transplant surgeon and contacted by the coordinator and will come into hospital for admission. The co-ordinator will request 3 units of packed red blood cells, matched to the intended recipient, for use in the OrganOx *metra*™ device. The liver will be prepared according to the procedure for preparing the device for use and placing the organ on the device (described in detail in the OrganOx *metra*™ Instructions for Use (IFU) document (version 13.0, 12-Mar-2016). The liver will be weighed prior to being connected to the device. If cannulation proves impossible, the liver will be rejected as previously intended. If the liver meets the criteria for transplantation, the recipient explantation will commence and the procedure for removing the liver from the device is also described in the IFU. Implantation and reperfusion of the liver will proceed as per the usual practice of the implanting centre. The patient will be clerked as if they were being admitted for a standard liver transplant.

Perfusion of the graft

The machine will be primed with a perfusate suitable for NMP-L and will use packed red cells as the oxygen carrier. During the perfusion, biochemical analysis of the blood-based perfusate will be performed using a Cobas biochemical point-of-care analyser (Roche Diagnostics) which will give results for pH, pO2, pCO2, Bicarb, Base excess, Calcium,

For peer review only

Chloride, Sodium, Potassium, Haemoglobin, Haematocrit, Lactate and Glucose. Arterial and portal venous flows, resistances and pressures will also be recorded. Samples to be collected are detailed in Table 2.

For peer review only

Table 2. Trial sample collection schedule.

Perfusate samples	Hepatic arterial and hepatic venous biochemistry (point-of-care)	Pre-perfusion<br>Every 30 minutes during perfusion	Cobas point-of-care desktop analyser
	Perfusate supernatant	Pre-perfusion<br>Every 15 mins for first hour<br>Every hour thereafter	5x1ml aliquots<br>Stored at -80°C
Liver samples	Liver biopsy	L1 Pre-perfusion<br>L2 After 4 hours<br>L3 at end of perfusion*<br>L4 Post-reperfusion	16G core needle biopsy<br>Divided into segment for formalin, segment for frozen and piece for electron microscopy.
	Common bile duct	CBD1 Pre-perfusion<br>CBD2 Post-reperfusion	Formalin
Bile samples	(if produced)	B2 sample at 2 hours<br>B4 sample at 4 hours<br>B6 sample at 6 hours	Total volume recorded and 2ml samples snap frozen at these time points
Patient Samples	Biochemistry<br>Haematology<br>Clotting	Visits 1, 2, 3, 4, Extended follow-up	Standard of care
	Serum, Plasma, mononuclear cells (PBMC)	Visit 1 (pre-operative [post-induction of anaesthesia], post-reperfusion<br>Day 4 post-op)<br>Visits 2, 3, 4,	Additional research samples
	Urine	Visit 1 (pre-operative [post-induction], post-reperfusion<br>Day 4 post-op)	Additional research samples

*if lasting longer than 6 hours

For peer review only

The duration of machine perfusion will be dictated by logistics and the recipient's explant, but should not be less than 4 hours or more than 24 hours. For a graft to be considered for transplantation it *must* –

- Metabolise lactate to less than or equal to 2.5mmol/L within 4 hours of the start of the perfusion

And meet at least 2 of the following criteria within 4 hours of the start of perfusion:

- Demonstrate evidence of bile production
- Maintain a pH greater than 7.30
- Show evidence of glucose metabolism
- Maintain stable hepatic arterial flow of more than or equal to 150 mL/ minute and portal flow more than or equal to 500 mL/minute
- Achieve homogeneous graft perfusion with soft consistency of the parenchyma

Once the transplanting surgeon is content that the liver has met the criteria required for transplantation, the recipient will be brought to theatre and the explant will commence.

Explantation, implantation and reperfusion of the liver will be carried out in using standardised techniques by the on-call transplant surgeon. The liver will remain on the machine until after the explantation has taken place at which point it will be flushed by 2L of cold HTK immediately prior to implantation.

Concomitant therapy/medications

Patients will receive immunosuppression according to hospital protocols and other medications as necessary for their co-morbidities and current clinical condition. Their post-operative care will be the same as if they had undergone a standard liver transplant.

Objectives and Outcome measures

Primary

There are 2 linked primary objectives and respective outcome measures:

Primary Objective: (A) - Establish the feasibility of NMP-L to increase the number of transplantable livers.

For peer review only

Primary Outcome measure: (A) – "Rescue rate" i.e. the proportion of rejected livers that can be used for transplantation having been deemed viable following a period of machine perfusion.

Primary Objective: (B) – To achieve successful transplantation of previously rejected donor livers following viability testing using NMP-L.

Primary Outcome Measure: (B) – 90-day patient survival, calculated as the number of patients alive 90-day post NMP-L treated marginal liver transplantation (numerator) divided by the total number of NMP-L treated marginal liver transplants performed (denominator).

Secondary

No hypotheses tests are planned for any secondary outcome measures, and analyses will be descriptive only.

The assessment of graft function post-transplantation by incidence of primary non-function and early allograft dysfunction will be carried out by comparing results with a contemporary matched recipient group of patients obtained from a prospectively maintained database, with adjustment for potential cofounders.

. The contemporary matched recipient group will be matched using the following:

- Patient Characteristics: Age, Sex, BMI, MELD, UKELD, Aetiology
- Donor Liver Characteristics: DCD or DBD, Sex

Secondary Objective (1) – Assessment of liver graft function following transplantation (by incidence of primary non-function, and early allograft dysfunction)

Secondary Outcome Measures (1) – Liver function tests; 90-day graft survival; 12-month patient; and graft survival.

Secondary Objective (2) – Assess morbidity associated with receipt of extended criteria graft that had previously been rejected.

Secondary Outcome Measures (2) – Adverse event rates and severity, graded according to the Clavien-Dindo classification[40] (Appendix 1); Requirement of renal replacement therapy; incidence of biliary complications (including incidence of ischemic type biliary lesions diagnosed on MRCP at 6 months); incidence of vascular complications; biopsy-proven acute rejection; reoperation rate; length of intensive therapy unit stay; and length of hospital stay.

For peer review only

Secondary Objective (3) – Assess the physiological response to reperfusion of the perfused grafts

Secondary Outcome Measures (3) – Post-reperfusion syndrome, defined as a decrease in mean arterial pressure (MAP) of more than 30% from the baseline value for more than one minute during the first five minutes after reperfusion (assessed in the context of inotrope use).

Secondary Objective (4) – Identify impact upon quality of life after transplantation with these liver grafts.

Secondary Outcome Measures (4) – Quality of life by delivery of the EQ-5D-5L questionnaire at baseline, day 30 and 6 months post-transplant.

Analytical methods

Histopathology

Two independent liver histopathologists from UHBFT will perform all the histopathological assessments. Both will be blinded to the graft type, and the primary and secondary outcome measures although the presence or absence of a post-reperfusion biopsy means they will know whether a graft has met the criteria for transplantation. The histological analysis will be established using haematoxylin and eosin at two levels as well as, periodic acid Schiff (PAS), periodic acid Schiff diastase (PASD), haematoxylin van Gieson (HVG), reticulin, orcein, rhodanin and Perls stains of formalin-fixed paraffin-embedded liver tissue.

Perfusion, clinical and laboratory data

Donor and patient demographics as well as intraoperative data will be collected. BMI was defined as weight in kilograms divided by the square of the height in metres (kg/m^2). In non-heart beating (DCD) donation, FWIT is defined as the time between the systolic blood pressure of the donor dropping below 50mmHg until the point of aortic perfusion. Cold ischemic time is defined as the time between aortic perfusion and the start of NMP-L. Donor risk index (DRI) and Balance of Risk (BAR) will be calculated as per the relevant literature.[11, 41]

The perfusate fluid will undergo point-of-care biochemical testing every 30 minutes as previously described. Perfusate will be taken at the time points described in Table 2 and tested for transaminase, urea, albumin and factor V levels. Patient's blood samples will be analysed for full blood count, urea, creatinine and electrolytes, liver function tests,

international normalised ratio (INR), prothrombin time, amylase, C reactive protein and plasma glucose using standard laboratory methods (Roche Modular system, Roche Ltd, Lewes, UK) both pre-and post-operatively. Research recipient blood and urine samples will also be taken as part of work package 2 (WP2) that will enable immune cell profiling as well as lipodomic, proteomic and metabolomic testing.

Patient questionnaires

Quality of life (QoL) will be assessed by delivery of the EQ-5D-5L questionnaire (UK (English) © 2009 EuroQol Group EQ-5D™ is a registered trademark of the EuroQol Group) at baseline, day 30 and 6 months' post-transplant. EQ-5D-5L is a 5-level version of the EQ-5D descriptive system (M. Herdman et al. Qual Life Res DOI 10.1007/s11136-011-99031). The 5L retains the 5-dimensional (5D) structure of the original EQ-5D-3L but the levels on each dimension were expanded to 5 based on qualitative and quantitative studies conducted by the EuroQol Group. Index-based values ('utilities') enable the calculation of quality-adjusted life years (QALYs) which help inform economic evaluations of health care interventions.

Statistical justification and outcome analysis

Sample size justification

For (A) feasibility of NMP-L to rescue discarded liver grafts, it is anticipated that NMP-L will achieve a desirable organ recovery rate of at least 50%, with an undesirable rate of 30% or less as this would not be considered economically feasible. The significance level (α) is set at 0.05, corresponding to the probability of incorrectly rejecting the hypothesis given it is true (Type I error), and the power is set at 0.90 (Type II error rate, $\beta = 0.10$), corresponding to the probability of correctly deciding the NMP-L treatment is successful given the true response rate is greater than 50%.

Using a Simon's two-stage design:[38]

Interim assessment stage 1A of accrual: 24 marginal grafts will be perfused and assessed in the first stage. Grafts will be transplanted depending on the criteria achieved. The procedure will be considered infeasible if there are fewer than 8 recovered livers. If more than 8 livers are transplanted, we will proceed to Stage 2A.

For peer review only

Final stage 2A of accrual: Up to additional 29 marginal grafts will be perfused. We would consider the procedure feasible if there are at least 22 recovered livers out of 53 perfused livers.

For (B) for viable livers transplanted following NMP-L, a desirable 90-day patient survival rate is at least 88%, with an undesirable rate of 73% (15% lower). The mean 90-day patient survival rate for 'standard' liver transplants is 93%.[42] An optimal three-stage design[39] will be used to test the null hypothesis that the mean 90-day patient survival rate will be less than 73% ($P \leq 0.73$), versus an alternative hypothesis - that the 90-day patient survival rate will be at least 88% ($P \geq 0.88$). The significance level is set at 0.20 (target $\alpha=0.2$), giving a 0.2 probability to conclude that a single transplantation is viable when it truly is not viable. The power is set at 80% (target $\beta=0.2$), giving a 0.2 probability to conclude that a single transplantation is not viable when it truly is viable.

Interim assessment stage 1B: Following transplantation in 3 patients, the trial will stop early (concluding $P \leq 0.73$) if there are fewer than 2 patients achieving 90-day survival. If 2 or more patients reach the primary end point of 90-day survival, an additional 8 transplantations will be performed.

Interim assessment stage 2B: Following transplantation in 11 patients (combined first and second stages) the trial will stop early (concluding $P \leq 0.73$) if there are seven or fewer successes. If 8 or more patients reach the primary end point, an additional 11 transplantations will be performed.

Final stage 3B: Following transplantation in 22 patients in all three stages, the trial will be successful if at least 18 patients reach the primary end-point of 90-day survival.

The trial schema is provided in Figure 2.

Analysis of outcome measures

Primary analysis

To assess (A) the feasibility of NMP-L, the rescue rate will be calculated as the number of perfused marginal grafts meeting the criteria for viability (numerator) divided by the total number of perfused marginal grafts (denominator).

$$Rescue\ Rate = \frac{Number\ of\ viable\ perfused\ marginal\ grafts}{Total\ number\ of\ perfused\ marginal\ grafts}$$

For peer review only

To assess (B) – achievement of successful transplantation of previously rejected donor liver following viability testing using NMP-L. We will evaluate 90-day patient survival rate, as an indicator of liver function and/or viability following transplantation of marginal liver grafts following NMP-L. The 90-day patient survival rate will be calculated as the number of patients alive at 90-day post-transplant with a VITTAL graft, divided by the total number VITTAL-patients transplanted.

$$90\ day\ patient\ survival\ rate = \frac{Number\ of\ patients\ alive\ at\ 90\ days\ post\ transplantation}{Total\ number\ of\ transplanations\ performed}$$

For (A), all livers undergoing NMP-L treatment will be included for evaluation in the interim and final analyses. For (B), all transplantations performed will be included for evaluation in the interim and final analyses. The rate outcomes will be reported together with confidence intervals using the Wilson (1927) method.[43]

Planned Interim Assessments

As we have utilised adaptive designs, there are planned formal interim assessments for both (A) feasibility of NMP-L and (B) successful transplantation of rescued livers, with clear "Go" / "No go" decisions as detailed earlier. Ideally, recruitment (i.e. transplantation) would stop whilst interim analyses of the primary outcome measures are performed. For (A) this could happen immediately, however for (B) this would result in a pause of over 3 months hence the pragmatic approach for such adaptive designs is to continue recruitment whilst they are being conducted.

To maximise patient safety, for (B) at the end of the first stage (transplantation of the first 3 patients), recruitment will be paused to allow the DMC to assess the initial safety data. Once all 3 patients are discharged, if the DMC considers the patients to be recovering well, with liver function that would be expected at this stage, recruitment can continue prior to the patients reaching the primary end-point of 90-day survival. A follow-on report will be sent to the DMC once the third patient reaches the primary endpoint. For the second stage (transplantation of 11 patients), safety data will be sent to the DMC for review after discharge of all 11 patients however recruitment need not stop at this point. A follow-on report will again be sent once the 11th patient reaches the primary endpoint.

For peer review only

DISCUSSION

The consequence of the escalating demand for liver transplantation is increasing waiting list mortality and in many countries, patients are more likely to die whilst waiting for an organ than in the first year after their transplant.[45] The outcomes of high-risk livers are inferior to standard grafts and the difference is most noticeable within the initial 90 days. Indeed, severe early allograft dysfunction or primary non-function often trigger post-transplant sepsis and multi-organ failure and as consequence, livers with marginal features are often declined and discarded.

Our preliminary experience and pilot transplant series showed NMP-L can provide objective information regarding liver function and the VITTAL trial aims to produce robust data and validate our initial observations.

Several challenges were identified when designing the VITTAL trial with the foremost being to create a sound definition of a discarded liver. There is an undeniable variation in utilisation of high-risk livers among the UK transplant centres which has been recognised and highlighted by NHSBT. The organisation published “Taking organ transplantation to 2020”, a strategy that aims to create greater consistency in the acceptance of organ offers and utilisation of marginal livers across all centers.[46] To address this issue for this study purposes, every declined liver offered for enrolment into VITTAL has to meet also at least one of a list of predefined, constant inclusion measures, adopted in combination with a two-consultant system of macroscopic liver quality assessment.

The most important factor to consider whilst designing a trial that pushes the current boundaries of high-risk livers utilisation is patient safety. Although we opted for liberal liver graft selection inclusion criteria, only low to moderate risk recipients are eligible to take part in this trial. Such an approach has been shown previously to be the safest and the most successful strategy for utilisation of high-risk organs.[47 48] The intended recipients will be risk stratified and selected by the Liver Unit’s liver transplant multi-disciplinary team. Another important trial safety feature is its three-stage adaptive design, introducing two interim safety analyses after completion of 3 and 11 transplants respectively.

For peer review only

There are undoubtedly some livers that will not be salvageable or ever safe to transplant. It is important for the purposes of the trial to include organs that fail to meet the defined viability criteria, to compare these with transplantable high-risk livers. The research work package linked with the trial was designed to identify sensitive point of care liver quality tests and propose novel biomarkers or panels associated with viable livers.

The primary end-point of 90-day patient survival has been chosen as it is a nationally accepted, monitored and continuously audited outcome following liver transplantation. Obviously, the graft survival rate is important and for the trial to truly be successful, patients who reach the primary end-point should have a VITTAL graft still *in-situ*. This will be considered when the DMC monitor the results at the interim analyses.

As well as the study design, challenges with trial logistics were also identified. One of the previously unseen difficulties after discussion with the haematology team, is the issuing of packed red cells matched to the intended recipient, potentially before the patient is admitted to hospital, to avoid delaying the start of the perfusion. When patients are listed, they undergo a blood cross-matching process to identify blood group and the presence of antibodies. This sample is not held for longer than 7 days by the hospital and so if they are admitted for a transplant or require blood products for some other intervention, they have a new sample sent before those products are issued. In the case of the VITTAL trial, a perfusion may need to commence before the patient is admitted to hospital as they may have to travel some distance. Minimising the cold ischemic time of marginal grafts is paramount to improve the chances of graft salvage. Therefore, in this scenario, blood is issued for the trial based on the results of the original sample and a repeat is sent when the patient is admitted to check they have not subsequently developed new antibodies. Blood product traceability is an important consideration and the blood products are documented to have been used in the device perfusate only and have not been used for recipient transfusion.

ETHICS AND DISSEMINATION

The VITTAL Clinical trial is an academic investigator-led study involving a CE marked medical device. The device is being used outside it current CE mark and therefore has been reviewed by the MHRA UK and received a “clinical investigation: no objection” (CI/2016/0031) letter : 3rd August 2016. In addition the study has undergone national ethical

For peer review only

review in the UK and received national ethical approval from the London – Dulwich Research ethics committee (16/LO/1056) and the Health Research Authority. In addition to the above national regulatory approvals the study has been reviewed by the National Health Service Blood and Transfusion service (NHSBT) and received all appropriate local institution/NHS R&D approvals . The trial management team are also fully engaged in an academic collaboration with the device manufacture Organox as part of the management of this study.

The trial management team are fully committed to publishing (within 12 months of the end of the study) the results of this study in accordance with best clinical practice in an open access, peer reviewed medical journal irrespective of outcome. Any dissemination of results, or publicity will be provided in a format which will not allow individual patients to be identified and confidentiality will be maintained throughout the process. The study management will be conducted in accordance with all applicable clinical trial regulations and managed centrally by the D3B trial management team – part of the CRUK clinical trials unit based in Birmingham, in accordance with the quality management system. The results of the study will also be made available directly to study participants and specialist patient groups.

SUMMARY

The presented VITTAL trial is the first clinical trial designed to objectively assess function of declined livers using NMP-L and subsequently transplanting viable grafts. It is hoped that the trial will identify a proportion of discarded organs that can be successfully transplanted and the generated data will provide objective and validated information that can be subsequently implemented in the process of acceptance and allocation of high-risk donor livers. This novel approach should improve consistency and increase utilisation of marginal liver grafts without compromising recipient safety.

For peer review only

ACKNOWLEDGMENTS, FINANCIAL SUPPORT AND SPONSORSHIP

This trial is funded by a grant awarded by the Wellcome Trust Health Innovation Challenge Fund (awarded in December 2015). The grant application was a joint effort of the co-applicant team (Darius Mirza, Hynek Mergental, Simon Afford, David Adams, Peter Friend, Stefan Hübscher, Julian Bion, Christina Yap) and collaborators (Paolo Muiesan, Thamara Perera, Hentie Cilliers, Ricky Bhogal, Richard Laing, Amanda Kirkham, James Ferguson, Desley Neil, Amanda Smith, Steven Youngs, Darren Barton and Leslie Russell). The project was endorsed and supported by all the clinical staff at the Liver Unit of UHBFT and we would like to thank everybody for their hard work and dedication to the transplant program.

RWL, YB and SCA have received support from the NIHR Birmingham Liver Biomedical Research Unit.

The views expressed in this publication are those of the authors and not necessarily those of the NHS, the National Institute for Health Research or the Department of Health.

AUTHORS' CONTRIBUTIONS

DFM (chief investigator) and HM (principal investigator) had the original concept of the VITTAL trial following on from a successful pilot study. CY (lead statistician), DFM, HM, AK (trial statistician) and RWL designed the VITTAL trial. RWL, MW, AK, CY and HM wrote the protocol. RWL, MW and DB completed the IRAS applications. DB, HM, AK, CY, RWL and DFM reviewed all protocol versions. RWL, MW and DB (senior trials co-ordinator) submitted all REC, MHRA, HRA, NHSBT and local R&D applications. AK, CY and DFM devised the statistical analysis plan. TP, PM, JI, KR, HM and DFM are the transplant surgeons involved in the trial. RWL and MW wrote the patient information sheets, external trial information and patient CRFs. RWL, HM and DFM wrote the manuscript and all authors reviewed the final version.

CONFLICT OF INTEREST

RWL and YB receive salary as Wellcome Trust research fellows.

For peer review only

Appendix 1. Definition of adverse events

ADVERSE EVENT (AE)

Any untoward medical occurrence in a subject.

NOTE this definition does not imply that there is a relationship between the adverse event and the device under investigation.

SERIOUS ADVERSE EVENT (SAE)

An adverse event that

a) led to a death,

b) led to a serious deterioration in the health of the subject that

1) resulted in a life-threatening illness or injury,

2) resulted in a permanent impairment of a body structure or a body function,

3) required in-patient hospitalization or prolongation of existing hospitalization,

4) resulted in medical or surgical intervention to prevent permanent impairment to body structure or a body function.

c) led to foetal distress, foetal death or a congenital abnormality or birth defect.

ADVERSE DEVICE EFFECT (ADE)

Any untoward and unintended response to a medical device.

NOTE 1 This definition includes any event resulting from insufficiencies or inadequacies in the instructions for use or the deployment of the device.

SERIOUS ADVERSE DEVICE EFFECT (SADE)

Adverse device effect that has resulted in any of the consequences characteristic of a serious adverse event or that might have led to any of these consequences if suitable action had not been taken or intervention had not been made or if circumstances had been less opportune.

UNANTICIPATED SERIOUS ADVERSE DEVICE EFFECTS (USADE)

Any serious adverse device effect which, by its nature, incidence, severity or outcome, has not been identified in the anticipated AE's listed in section 8.1.1.

DEVICE DEFICIENCY

For peer review only

Inadequacy of a medical device with respect to its identity, quality, durability, reliability, safety or performance. Device deficiencies include malfunctions, use errors and inadequate labelling.

USE ERROR

Act or omission of an act that results in a different medical device response than intended by the manufacturer or expected by the user. Use error includes slips, lapses and mistakes. An unexpected physiological response of the subject does not itself constitute a use error.

SEVERITY DEFINITIONS

The following definitions will be used to determine the severity rating for all adverse events:
Mild: awareness of signs or symptoms, that does not interfere with the subject's usual activity or is transient that resolved without treatment and with no sequelae.
Moderate: a sign or symptom, which interferes with the subject's usual activity.
Severe: incapacity with inability to do work or perform usual activities.

For peer review only

Clavien Dindo Classification

Grades	*Definition*
Grade I:	Any deviation from the normal postoperative course without the need for pharmacological treatment or surgical, endoscopic and radiological interventions. This grade also includes wound infections opened at the bedside. [Allowed therapeutic regimens are: drugs as anti-emetics, antipyretics, analgesics, diuretics and electrolytes and physiotherapy.]
Grade II:	Requiring pharmacological treatment with drugs (other than those noted above) including blood transfusions and total parenteral nutrition
Grade III:	Requiring surgical, endoscopic or radiological intervention
Grade III-a:	intervention not under general anaesthesia
Grade III-b:	intervention under general anaesthesia
Grade IV:	Life-threatening complication (including CNS complications)[1] requiring HDU/ICU management
Grade IV-a:	single organ dysfunction (includes dialysis)
Grade IV-b:	multi organ dysfunction
Grade V:	Death of a patient
Suffix 'd':	If the patients suffers from a complication at the time of discharge, the suffix "d" (for 'disability') is added to the respective grade of complication.

[1]brain haemorrhage, ischaemic stroke, subarachnoid haemorrhage, but excluding transient ischaemic attacks; HDU, high dependency unit; ICU, intensive care unit.

For peer review only

References

1. Williams R, Aspinall R, Bellis M, et al. Addressing liver disease in the UK: a blueprint for attaining excellence in health care and reducing premature mortality from lifestyle issues of excess consumption of alcohol, obesity, and viral hepatitis. *Lancet* 2014;384(9958):1953-97. doi: 10.1016/S0140-6736(14)61838-9

2. Williams R, Ashton K, Aspinall R, et al. Implementation of the Lancet Standing Commission on Liver Disease in the UK. *Lancet* 2015;386(10008):2098-111. doi: 10.1016/S0140-6736(15)00680-7

3. Mathur AK, Ranney DN, Patel SP, et al. The effect of smoking on biliary complications following liver transplantation. *Transpl Int* 2011;24(1):58-66. doi: 10.1111/j.1432-2277.2010.01146.x [published Online First: 2010/08/26]

4. NHS Blood and Transplant. Organ Donation and Transplantation Activity Report 2015/16, 2016.

5. NHS Blood and Transplant. Annual report on liver transplanation 2015/16, 2016.

6. Sotiropoulos GC, Radtke A, Molmenti EP, et al. Long-term follow-up after right hepatectomy for adult living donation and attitudes toward the procedure. *Ann Surg* 2011;254(5):694-700; discussion 00-1. doi: 10.1097/SLA.0b013e31823594ae

7. Simoes P, Kesar V, Ahmad J. Spectrum of biliary complications following live donor liver transplantation. *World J Hepatol* 2015;7(14):1856-65. doi: 10.4254/wjh.v7.i14.1856

8. Mirza DF, Gunson BK, Da Silva RF, et al. Policies in Europe on "marginal quality" donor livers. *Lancet* 1994;344(8935):1480-3.

9. NHS Blood and Transplant. Organ Donation and Transplantation Activity Report 2014/15, 2015.

10. Working Party of The British Transplantation Society. Transplantation from donors after deceased circulatory death, 2013.

11. Feng S, Goodrich NP, Bragg-Gresham JL, et al. Characteristics associated with liver graft failure: the concept of a donor risk index. *Am J Transplant* 2006;6(4):783-90.

12. Braat AE, Blok JJ, Putter H, et al. The Eurotransplant donor risk index in liver transplantation: ET-DRI. *Am J Transplant* 2012;12(10):2789-96. doi: 10.1111/j.1600-6143.2012.04195.x

13. Belzer FO, Southard JH. Principles of solid-organ preservation by cold storage. *Transplantation* 1988;45(4):673-6. [published Online First: 1988/04/01]

14. Carini R, Autelli R, Bellomo G, et al. Alterations of cell volume regulation in the development of hepatocyte necrosis. *Exp Cell Res* 1999;248(1):280-93. doi: 10.1006/excr.1999.4408

For peer review only

15. Pratschke J, Weiss S, Neuhaus P, et al. Review of nonimmunological causes for deteriorated graft function and graft loss after transplantation. *Transpl Int* 2008;21(6):512-22. doi: 10.1111/j.1432-2277.2008.00643.x

16. Menger MD, Pelikan S, Steiner D, et al. Microvascular ischemia-reperfusion injury in striated muscle: significance of "reflow paradox". *Am J Physiol* 1992;263(6 Pt 2):H1901-6.

17. Vogel T, Brockmann JG, Coussios C, et al. The role of normothermic extracorporeal perfusion in minimizing ischemia reperfusion injury. *Transplant Rev (Orlando)* 2012;26(2):156-62. doi: 10.1016/j.trre.2011.02.004

18. Henry SD, van der Wegen P, Metselaar HJ, et al. Hydroxyethyl starch-based preservation solutions enhance gene therapy vector delivery under hypothermic conditions. *Liver Transpl* 2008;14(12):1708-17. doi: 10.1002/lt.21623

19. Mediavilla MG, Krapp A, Carrillo N, et al. Efficient cold transfection of pea ferredoxin-NADP(H) oxidoreductase into rat hepatocytes. *J Gene Med* 2006;8(3):306-13. doi: 10.1002/jgm.847

20. Ke B, Shen XD, Tsuchihashi S, et al. Viral interleukin-10 gene transfer prevents liver ischemia-reperfusion injury: Toll-like receptor-4 and heme oxygenase-1 signaling in innate and adaptive immunity. *Hum Gene Ther* 2007;18(4):355-66. doi: 10.1089/hum.2007.181

21. Le Moine O, Louis H, Demols A, et al. Cold liver ischemia-reperfusion injury critically depends on liver T cells and is improved by donor pretreatment with interleukin 10 in mice. *Hepatology* 2000;31(6):1266-74. doi: 10.1053/jhep.2000.7881

22. Ambiru S, Uryuhara K, Talpe S, et al. Improved survival of orthotopic liver allograft in swine by addition of trophic factors to University of Wisconsin solution. *Transplantation* 2004;77(2):302-19. doi: 10.1097/01.TP.0000100468.94126.AF

23. Koti RS, Seifalian AM, Davidson BR. Protection of the liver by ischemic preconditioning: a review of mechanisms and clinical applications. *Dig Surg* 2003;20(5):383-96. doi: 72064

24. Raeburn CD, Zimmerman MA, Arya J, et al. Ischemic preconditioning: fact or fantasy? *J Card Surg* 2002;17(6):536-42.

25. Oniscu GC, Randle LV, Muiesan P, et al. In situ normothermic regional perfusion for controlled donation after circulatory death-the United kingdom experience. *Am J Transplant* 2014;14(12):2846-54. doi: 10.1111/ajt.12927

26. Ravikumar R, Jassem W, Mergental H, et al. Liver transplantation after ex vivo normothermic machine preservation: a Phase 1 (first-in-man) clinical trial. *Am J Transplant* 2016 doi: 10.1111/ajt.13708

27. Tuttle-Newhall JE, Collins BH, Kuo PC, et al. Organ donation and treatment of the multi-organ donor. *Curr Probl Surg* 2003;40(5):266-310. doi: 10.1016/S0011-3840(03)00011-X

For peer review only

28. Frost AE. Donor criteria and evaluation. *Clin Chest Med* 1997;18(2):231-7.

29. Pirenne J, Monbaliu D, Van Gelder F, et al. Liver transplantation using livers from septuagenarian and octogenarian donors: an underused strategy to reduce mortality on the waiting list. *Transplant Proc* 2005;37(2):1180-1. doi: 10.1016/j.transproceed.2004.12.168

30. Biancofiore G, Bindi M, Ghinolfi D, et al. Octogenarian donors in liver transplantation grant an equivalent perioperative course than ideal young donors. *Dig Liver Dis* 2017 doi: 10.1016/j.dld.2017.01.149

31. Ferla F, De Carlis R, Mariani A, et al. Liver transplant using octogenarian donors. *Liver Transpl* 2016;22(7):1040-1. doi: 10.1002/lt.24450

32. Hellinger A, Fiegen R, Lange R, et al. Preservation of pig liver allografts after warm ischemia: normothermic perfusion versus cold storage. *Langenbecks Archiv fur Chirurgie* 1997;382(4):175-84.

33. Schon MR, Kollmar O, Wolf S, et al. Liver transplantation after organ preservation with normothermic extracorporeal perfusion. *Ann Surg* 2001;233(1):114-23.

34. Imber CJ, St Peter SD, Lopez de Cenarruzabeitia I, et al. Advantages of normothermic perfusion over cold storage in liver preservation. *Transplantation* 2002;73(5):701-9. [published Online First: 2002/03/22]

35. Butler AJ, Rees MA, Wight DG, et al. Successful extracorporeal porcine liver perfusion for 72 hr. *Transplantation* 2002;73(8):1212-8.

36. Brockmann J, Reddy S, Coussios C, et al. Normothermic perfusion: a new paradigm for organ preservation. *Ann Surg* 2009;250(1):1-6. doi: 10.1097/SLA.0b013e3181a63c10

37. Perera M, Mergental H, Stephenson B, et al. First human liver transplantation using a marginal allograft resuscitated by normothermic machine perfusion. *Liver Transplant* 2015 doi: 10.1002/lt.24369

38. Simon R. Optimal two-stage designs for phase II clinical trials. *Control Clin Trials* 1989;10(1):1-10.

39. Chen TT. Optimal three-stage designs for phase II cancer clinical trials. *Stat Med* 1997;16(23):2701-11.

40. Dindo D, Demartines N, Clavien PA. Classification of surgical complications: a new proposal with evaluation in a cohort of 6336 patients and results of a survey. *Ann Surg* 2004;240(2):205-13. [published Online First: 2004/07/27]

41. Dutkowski P, Oberkofler CE, Slankamenac K, et al. Are there better guidelines for allocation in liver transplantation? A novel score targeting justice and utility in the model for end-stage liver disease era. *Ann Surg* 2011;254(5):745-53; discussion 53. doi: 10.1097/SLA.0b013e3182365081

42. NHSBT. Activity report 2014/15, 2014/15.

For peer review only

43. Wilson EB. Probable Inference, the Law of Succession, and Statistical Inference. . *JASA* 1927;22(158):209-12.

44. Dutkowski P, Schlegel A, Slankamenac K, et al. The use of fatty liver grafts in modern allocation systems: risk assessment by the balance of risk (BAR) score. *Ann Surg* 2012;256(5):861-8; discussion 68-9. doi: 10.1097/SLA.0b013e318272dea2

45. NHSBT, NHSEngland. Annual Report on Liver Transplantation: Report for 2015/2016. September 2016 [published Online First: Sept 2016]

46. Walker KG, Eastmond CJ, Best PV, et al. Eosinophilia-myalgia syndrome associated with prescribed L-tryptophan. *Lancet* 1990;336(8716):695-6.

47. Avolio AW, Halldorson JB, Burra P, et al. Balancing utility and need by means of donor-to-recipient matching: a challenging problem. *Am J Transplant* 2013;13(2):522-3. doi: 10.1111/ajt.12031

48. Dutkowski P, Oberkofler CE, Slankamenac K, et al. Are there better guidelines for allocation in liver transplantation? A novel score targeting justice and utility in the model for end-stage liver disease era. *Ann Surg* 2011;254(5):745-53; discussion 53. doi: 10.1097/SLA.0b013e3182365081 [published Online First: 2011/11/02]

For peer review only

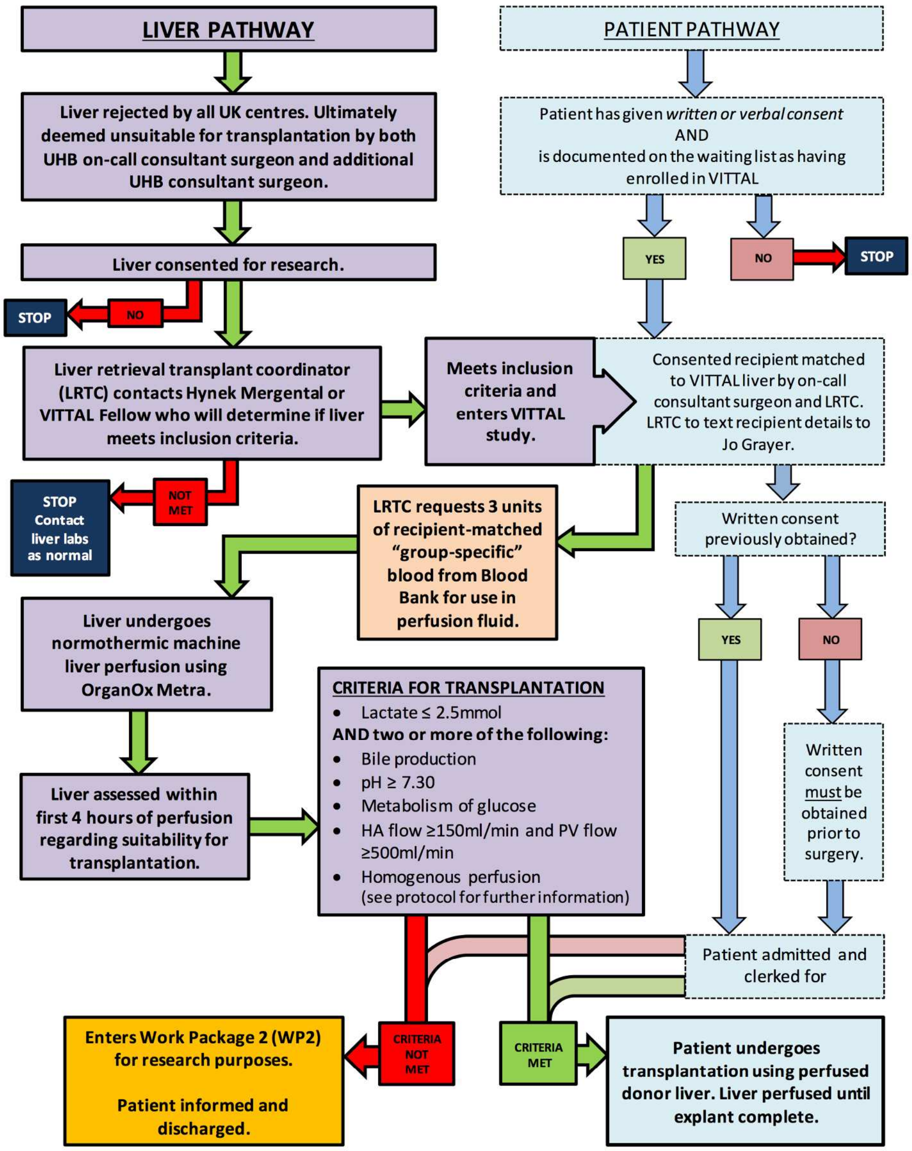

Figure 1. Patient and donor liver pathways

179x230mm (300 x 300 DPI)

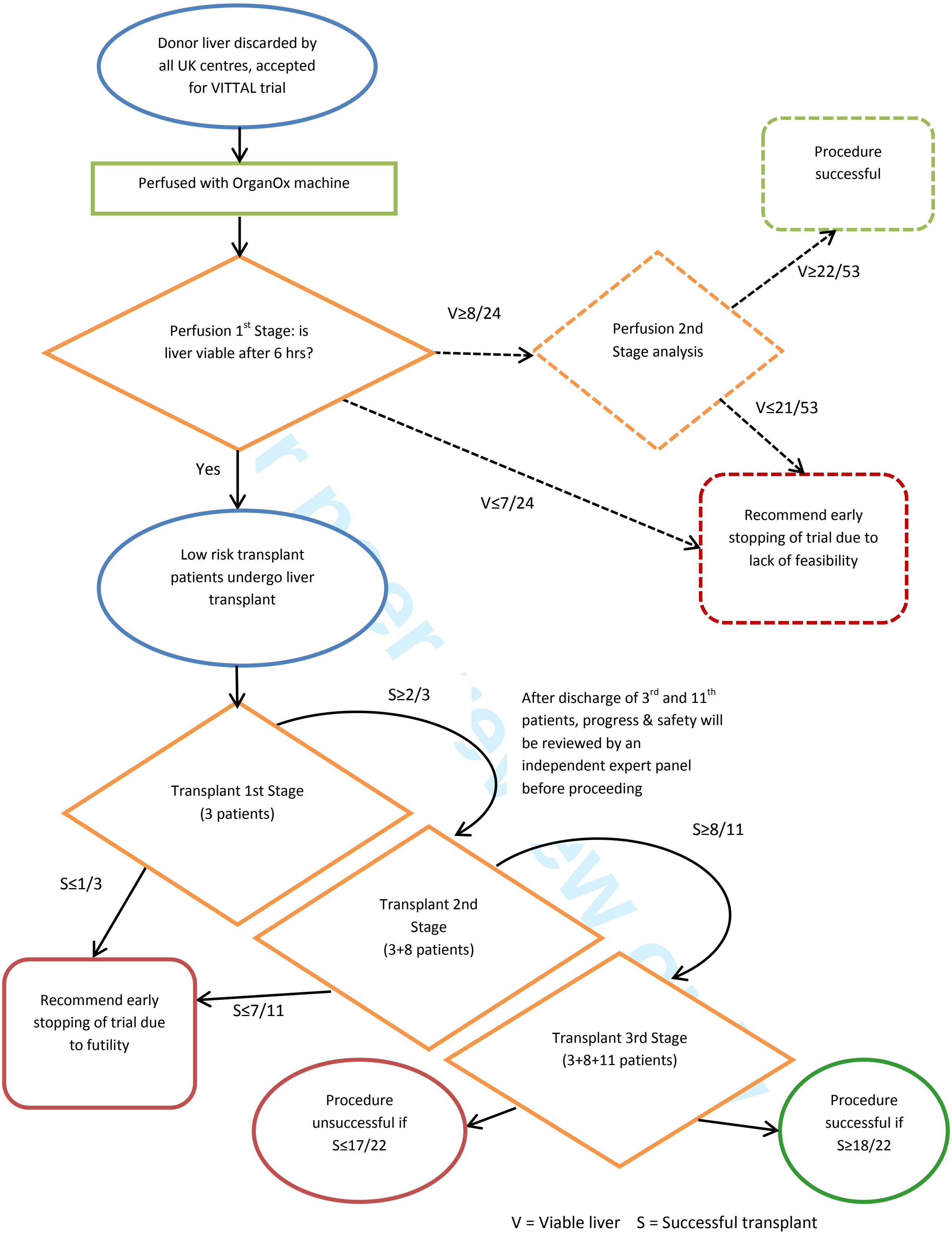

Figure 2: Trial Schema
